# U-Shaped Lipid-CVD Links in Older Adults: Reversed LDL-C and HDL-C Associations and Goldilocks Zone

**DOI:** 10.1016/j.jacadv.2025.102544

**Published:** 2026-01-22

**Authors:** Xiaoxin Ye, Shengyan Du, Shimin Chen, Yueting Shi, Wenchang Wang, Yanhao Wan, Jianhua Wang, Jing Zeng, Chunjiang Pan, Shanshan Yang, Miao Liu, Shengshu Wang, Yao He

**Affiliations:** a Medical School of Chinese People’s Liberation Army, Beijing, China; b Institute of Geriatrics, Beijing Key Laboratory of Geriatric Comorbidity, National Clinical Research Center for Geriatrics Diseases, Second Medical Center, Chinese People's Liberation Army General Hospital, Beijing, China; c Department of Anti-Nuclear, Biological, Chemical Medicine, Graduate School, Chinese People’s Liberation Army General Hospital, Beijing, China; d Ninth Medical Center, Chinese People's Liberation Army General Hospital, Beijing, China; e Department of Field Internal Medicine, Graduate School of Medical College of Chinese People's Liberation Army General Hospital, Beijing, China; f State Key Laboratory of Kidney Diseases, Beijing, China

**Keywords:** cohort study, CVD morbidity, CVD mortality, high-density lipoprotein cholesterol, low-density lipoprotein cholesterol, older adults

## Abstract

**Background:**

Guidelines and consensus statements lack consistent management targets and ranges for low density lipoprotein cholesterol (LDL-C) and high density lipoprotein cholesterol (HDL-C) in older adults.

**Objectives:**

The objectives of the study were to investigate the relationships of LDL-C and HDL-C with cardiovascular disease (CVD) risk in older adults and to identify optimal target ranges for their management.

**Methods:**

This study included 217,442 U.K. Biobank participants aged ≥60 years, free of CVD and cancer at baseline. Multivariable Cox regression models and restricted cubic splines were employed to estimate HRs and 95% CIs for the associations of LDL-C and HDL-C levels with CVD outcome.

**Results:**

During follow-up, 26,756 CVDs and 2,726 CVD deaths occurred. LDL-C exhibited U-shaped relationships with both incident CVD and CVD mortality (minimal risk: 3.600-4.204 mmol/L). HDL-C showed an L-shaped association with incident CVD but a U-shaped association with CVD mortality (minimal risk: 1.421-1.699 mmol/L). Compared with the reference LDL-C group, the Q1 group had a 39% higher incident CVD risk (HR: 1.39; 95% CI: 1.32-1.47) and 27% higher CVD mortality risk (HR: 1.27; 95% CI: 1.08-1.48). Conversely, compared with the reference HDL-C group, the Q6 group was associated with a lower incident CVD (HR: 0.90; 95% CI: 0.84-0.97) and higher CVD mortality risk (HR: 1.24; 95% CI: 1.01-1.51). Notably, the CVD risk rose progressively with joint LDL-C and HDL-C risk tiers.

**Conclusions:**

U/L-shaped associations of LDL-C and HDL-C with CVD risk in older adults underscore the importance of maintaining lipids within an optimal range. This distinct “Goldilocks zone” is associated with minimized risk and highlights current guideline limitations.

Guidelines and consensus statements lack consistent management targets and ranges for low-density lipoprotein cholesterol (LDL-C) and high-density lipoprotein cholesterol (HDL-C) in older populations. The 2019 European Atherosclerosis Society/European Society of Cardiology dyslipidemia guidelines recommend that LDL-C targets may be relaxed for older individuals with limited life expectancy (<1-2 years) or severe frailty while cautioning against 2 extremes: overtreatment and undertreatment.^1^ Conversely, the 2018 American College of Cardiology/American Heart Association guidelines emphasize percentage-based LDL-C reduction (≥50% for very high-risk patients) over absolute targets.^2^ Similarly, China's 2023 Lipid Management Guidelines highlight that low LDL-C predominates older Chinese populations, setting an LDL-C target of <1.8 mmol/L for very high-risk groups.^3^

Notably, no guideline currently establishes specific HDL-C targets, although all acknowledge low HDL-C as an independent atherosclerotic cardiovascular disease (CVD) (ASCVD) risk factor.^4^ Across all strategies, LDL-C reduction through lifestyle modifications and statin-based therapy remains the primary intervention despite the under-representation of older populations in pivotal clinical trials.^5^

Current guidelines broadly recognize LDL-C as a causal risk factor for CVD, whereas HDL-C is considered vasculoprotective through mechanisms such as reverse cholesterol transport.^6^, ^7^, ^8^ However, the foundational assumption that these “bad” and “good” cholesterol levels exhibit monotonic linear relationships with CVD risk—whether independently or synergistically—and the clinically optimal target ranges for these lipids remain scientifically elusive in older populations.^9^^,^^10^

Amidst global demographic aging, the proportion of individuals aged ≥60 years is projected to reach 21.1% worldwide by 2050. As CVD remains the leading cause of mortality within this demographic, its escalating burden necessitates urgent intervention.^11^ Primary prevention targeting lipid-attributable CVD risks thus holds substantial public health significance. However, current clinical guidelines exhibit critical evidence gaps regarding optimal lipid targets and management ranges for older adults, largely due to limited high-quality studies and inconsistent existing evidence. Leveraging the extensive U.K. Biobank (UKB) cohort, this study re-evaluates both individual and synergistic associations of LDL-C and HDL-C with CVD risk in older populations, quantifying safety thresholds for lipid management to deliver evidence-based epidemiological guidance for geriatric cardiovascular prevention.

## Methods

### Study design and population

**Figure 1:**
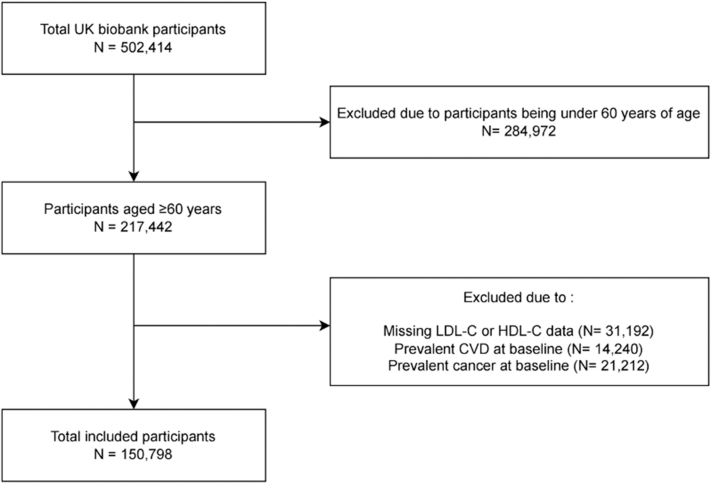
**Selection of U.K. Biobank Participants** CVD = cardiovascular disease; HDL-C = high-density lipoprotein cholesterol; LDL-C = low-density lipoprotein cholesterol.

Between 2006 and 2010, UKB enrolled 502,413 participants aged 40 to 73 years through primary care registries.^12^ Standardized protocols for data collection and biochemical assays (eg, lipid profiling) are detailed in the study’s technical documentation. For the current study, participants aged ≥60 years (n = 217,442) were considered. Individuals were excluded if they had missing LDL-C or HDL-C data (n = 31,192) or had prevalent CVD (n = 14,240) or cancer (n = 21,212) at baseline. Ultimately, 150,798 participants were included in the analysis and further categorized into 6 subgroups based on LDL-C and HDL-C percentiles (5th, 25th, 50th, 75th, and 95th) (Figure 1).

### Data collection and definition

Physical measurements, lifestyle factors, medical and family histories, and laboratory biomarkers were assessed at baseline and all subsequent follow-up visits using International Standardization Program.^12^ Participant age was defined as at enrollment into the cohort and categorized into 2 groups (<65 and ≥65 years) for subgroup analyses. Sex information, derived from National Health Service registration records at recruitment, was used without modification. Race was classified as White or other. Alcohol consumption and smoking status were defined as binary variables (never vs past/current use). Hypertension diagnosis required fulfillment of at least 1 of the following criterion: 1) self-reported physician-diagnosed hypertension; 2) self-reported antihypertensive medication use; 3) systolic blood pressure ≥140 mm Hg; or 4) diastolic blood pressure ≥90 mm Hg (Supplemental Table 1). Diabetes diagnosis required at least 1 of the following criterion: 1) self-reported physician-diagnosed diabetes; 2) self-reported glucose-lowering medication use, 3) fasting blood glucose ≥7.0 mmol/L; or 4) glycated hemoglobin ≥6.5% (48 mmol/L) (Supplemental Table 1). Polygenic risk scores (PRS) for HDL-C and LDL-C were obtained from the UKB-standardized PRS repository. Genetic predisposition was stratified as high (quintile 5), intermediate (quintiles 2-4), or low (quintile 1).

The study outcomes comprised incident CVD and CVD mortality. CVD encompassed ischemic heart disease, ischemic stroke, and hemorrhagic stroke (HS), identified through linkage to hospital inpatient records. Participants without baseline CVD were followed from enrollment until incident diagnosis, death, or November 1, 2022 (censor date). CVD events were defined using International Classification of Diseases, 10th Revision codes (Supplemental Table 2).

### Statistical analysis

All statistical analyses were performed using RStudio (version 4.2.3; Posit Software, PBC [formerly RStudio, Inc]). Two-tailed *P* values <0.05 were considered statistically significant. Continuous variables are presented as mean ± SD or median (25th-75th percentiles [Q1-Q3]) as appropriate. Baseline characteristics were compared using analysis of variance for normally distributed data and the Kruskal-Wallis test for non-normally distributed data. Categorical variables are expressed as frequencies (percentages) and were compared using the Pearson chi-square or Fisher exact test based on expected cell count.

Missing data were imputed using multiple imputation by chained equations, with covariate selection for imputation models optimized by random forest (RF). RF identified top predictive covariates for each incomplete variable (based on Gini importance), and 10 imputed data sets were generated (20 iterations/chain, convergence confirmed by trace plots). Pooled estimates were derived using Rubin rules to integrate results across imputed data sets. (see Supplemental Table 3 for missingness proportions).

Dose-response relationships between LDL-C and HDL-C with all endpoints were evaluated using restricted cubic splines (RCSs) with 3 to 5 knots placed at clinically relevant percentiles, incorporated into multivariable Cox proportional hazards models. We systematically evaluated penalized splines, RCS, natural splines, and fractional polynomials through theoretical derivation and simulation validation.^13^ The optimal number of knots was determined by minimizing the Akaike Information Criterion to balance model fit and overfitting. Multicollinearity was assessed using variance inflation factors (<2.0 for all covariates). Covariate selection was guided by clinical expertise, change in effect size value, Spearman's correlation matrix, and univariable Cox regression and supplemented by the Boruta algorithm based on RFs to evaluate variable importance (Supplemental Figures 1 to 3, Supplemental Tables 5 and 6).

Cox proportional hazard regression models were constructed to estimate the HR and 95% CI for associations between LDL-C and HDL-C subgroups defined by RCS inflection points and risk of CVD. In these primary models, participants who died from non-CVD causes were censored at their date of death. This approach is aimed at evaluating the biological effect of the exposure on the hazard of the event of interest. The proportional hazards assumption was verified using Schoenfeld residual tests. Model 1 adjusted for sex and age; model 2 additionally adjusted for race, alcohol consumption, smoking status, hypertension, diabetes, and body mass index. The Goldilocks zone is hypothesized as a value range capturing inflectional thresholds in the association between LDL-C and HDL-C level with CVD risk, derived from 6 distinct percentile-stratified subgroups.

**Figure 2:**
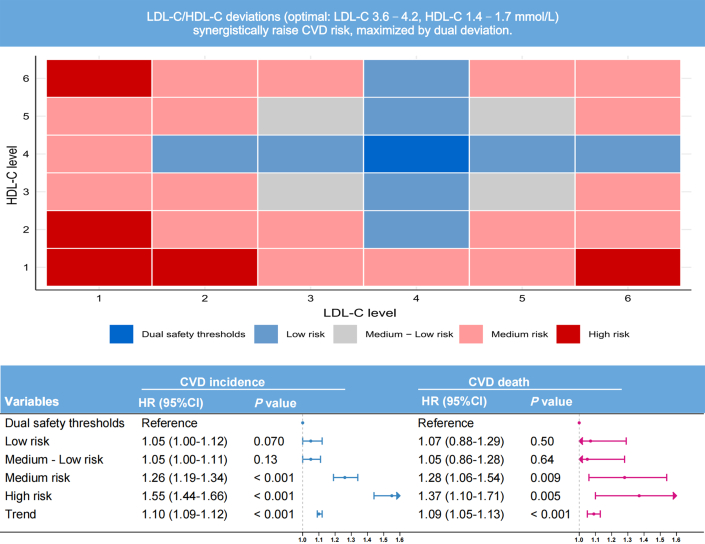
**Risk Stratification by Combined Lipid Profile** Model adjusted for sex, age, race, alcohol consumption, smoking status, hypertension, diabetes, and BMI. Abbreviations as in Figure 1.

A risk stratification system was developed by integrating the combined deviations of LDL-C and HDL-C levels from reference subgroups defined by RCS inflection points. Participants were categorized as: 1) reference: both lipids in reference subgroups; 2) low-risk: one lipid in reference subgroup and cumulative deviation (sum of both deviations) ≤2; 3) moderate-low-risk: both lipids nonreference and cumulative deviation ≤2; (4) moderate-risk: cumulative deviation 3 to 4; and 5) high-risk: cumulative deviation >4 (Figure 2). Subgroup analyses assessed heterogeneity across age (<65 vs ≥ 65 years), sex (male vs female), lipid-lowering medication use (yes vs no), and genetic predisposition (high vs medium vs low). Sensitivity analyses excluding participants who died within 1, 3, and 5 years of follow-up were performed to evaluate the robustness of our findings. To explicitly account for the competing risk of non-CVD death, we performed a supplementary analysis using Fine-Gray subdistribution hazards models.

UKB received ethical clearance from the North West Multi-Centre Research Ethics Committee (REC: 11/NW/0382). And all individuals gave written consent before taking part.

## Results

### Participant characteristics

Table 1 presents the baseline characteristics of included 150,798 UKB participants, with a median age of 64 (Q1-Q3: 62-66) years, and 46.8% males. Compared to participants without CVD death events (n = 148,072), those who developed CVD death (n = 2,726) were older, had lower educational attainment, lower socioeconomic status, more current or former smokers, consume alcohol less frequently, and report reduced physical activity. In addition, this group exhibited higher body mass index, C-reactive protein, glycated hemoglobin , waist circumference, systolic blood pressure, diastolic blood pressure, and triglyceride levels, while demonstrating lower HDL-C and LDL-C concentrations. Prevalent hypertension and diabetes, use of lipid-lowering medications, and family history of CVD were also more common among participants with CVD events.

**Table 1:** Baseline Characteristics of 150,798 Individuals

Characteristics	Overall (N = 150,798)	Non-CVD Death Event (n = 148,072)	CVD Death (n = 2,726)
Age, y	64.0 (62.0-66.0)	64.0 (62.0-66.0)	65.0 (63.0-67.0)
White race	140,241 (93.0)	137,723 (93.0)	2,518 (92.4)
Male	70,643 (46.9)	68,820 (46.5)	1,823 (66.9)
Body mass index, kg/m^2^	26.9 (24.5-29.8)	26.9 (24.4-29.8)	27.8 (25.1-31.0)
Blood glucose, mmol/L	5.0 (4.7-5.4)	5.0 (4.7-5.4)	5.1 (4.7-5.6)
C-reactive protein, mg/L	1.5 (0.8-3.0)	1.5 (0.8-3.0)	2.1 (1.0-4.1)
Diastolic blood pressure, mm Hg	82.5 (76.0-89.0)	82.5 (76.0-89.0)	83.5 (76.5-91.0)
High-density lipoprotein cholesterol, mmol/L	1.4 (1.2-1.7)	1.4 (1.2-1.7)	1.3 (1.1-1.6)
Glycated hemoglobin, mmol/mol	36.1 (33.9-38.6)	36.1 (33.9-38.6)	37.3 (34.6-40.8)
Low-density lipoprotein cholesterol, mmol/L	3.6 (3.0-4.2)	3.6 (3.0-4.2)	3.4 (2.7-4.0)
Systolic blood pressure, mm Hg	143.0 (131.0-155.5)	142.5 (131.0-155.5)	147.8 (135.5-162.0)
Total cholesterol, mmol/L	5.8 (5.0-6.6)	5.8 (5.0-6.6)	5.4 (4.6-6.3)
Triglycerides, mmol/L	1.6 (1.1-2.2)	1.6 (1.1-2.2)	1.7 (1.2-2.3)
Waist circumference, cm	91.0 (82.0-100.0)	91.0 (82.0-100.0)	96.0 (88.0-105.0)
Low-density lipoprotein cholesterol polygenic risk score, no unit	−0.06 (−0.75 to 0.60)	−0.06 (−0.75 to 0.60)	−0.05 (−0.75 to 0.64)
High-density lipoprotein cholesterol polygenic risk score, no unit	0.02 ± 1.01	0.02 ± 1.01	−0.02 ± 1.03
Albumin, g/L	44.9 (43.2-46.5)	44.9 (43.2-46.5)	44.5 (42.7-46.2)
Educational level			
Below high school	97,210 (64.5)	95,307 (64.4)	1,903 (69.8)
High school	13,577 (9.0)	13,365 (9.0)	212 (7.8)
Postsecondary education	40,011 (26.5)	39,400 (26.6)	611 (22.4)
Townsend Deprivation Index lever			
Low	53,443 (35.4)	52,669 (35.6)	774 (28.4)
Low - medium	46,732 (31.0)	45,962 (31.0)	770 (28.2)
Medium	23,607 (15.7)	23,159 (15.6)	448 (16.4)
Medium - high	15,683 (10.4)	15,301 (10.3)	382 (14.0)
High	11,333 (7.5)	10,981 (7.4)	352 (12.9)
International Physical Activity Questionnaire level			
Low	21,991 (14.6)	21,476 (14.5)	515 (18.9)
Medium	65,070 (43.2)	63,946 (43.2)	1,124 (41.2)
High	63,737 (42.3)	62,650 (42.3)	1,087 (39.9)
Current/former smoker	73,696 (48.9)	72,011 (48.6)	1,685 (61.8)
Current/former drinker	143,760 (95.3)	141,188 (95.4)	2,572 (94.4)
Lipid-lowering drugs	35,435 (23.5)	34,443 (23.3)	992 (36.4)
Family history of cardiovascular disease	61,898 (41.1)	60,751 (41.0)	1,147 (42.1)
Diabetes	12,922 (8.6)	12,412 (8.4)	510 (18.7)
Hypertension	103,262 (68.5)	101,035 (68.2)	2,227 (81.7)
Low-density lipoprotein cholesterol level, mmol/L			
Q1	7,550 (5.0)	7,297 (4.9)	253 (9.3)
Q2	30,152 (20.0)	29,476 (19.9)	676 (24.8)
Q3	37,752 (25.0)	37,082 (25.0)	670 (24.6)
Q4	37,672 (25.0)	37,109 (25.1)	563 (20.7)
Q5	30,142 (20.0)	29,697 (20.1)	445 (16.3)
Q6	7,530 (5.0)	7,411 (5.0)	119 (4.4)
High-density lipoprotein cholesterol level, mmol/L			
Q1	7,611 (5.1)	7,337 (5.0)	274 (10.1)
Q2	30,136 (20.0)	29,366 (19.8)	770 (28.2)
Q3	37,776 (25.1)	37,097 (25.1)	679 (24.9)
Q4	37,602 (24.9)	37,063 (25.0)	539 (19.8)
Q5	30,148 (20.0)	29,785 (20.1)	363 (13.3)
Q6	7,525 (5.0)	7,424 (5.0)	101 (3.7)

Values are median (25th–75th percentiles), mean ± SD, or n (%). ∗The “non-CVD death” group contains patients who had not experienced a CVD death before their last known follow-up time (censoring).

Low-density lipoprotein cholesterol level Q1: 0.276 to 2.211, Q2: 2.211 to 3.000, Q3: 3.000 to 3.600, Q4: 3.600 to 4.204, Q5: 4.204 to 5.145, Q6: 5.145 to 9.610. High-density lipoprotein cholesterol level Q1: 0.252 to 0.935, Q2: 0.935 to 1.191, Q3: 1.191 to 1.421, Q4: 1.421 to 1.699, Q5: 1.699 to 2.174, Q6: 2.174 to 4.107.

### Association between LDL-C and CVD outcomes

**Figure 3:**
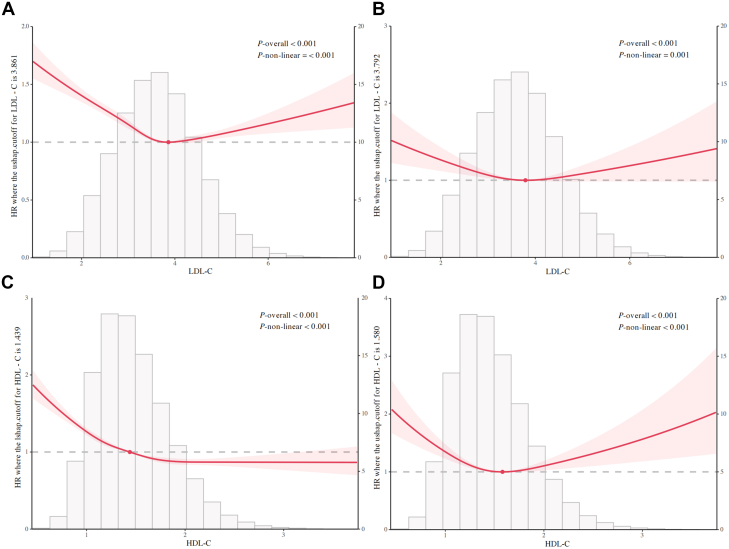
**Nonlinear Associations of Lipid Profile With Cardiovascular Disease Risk** (A) LDL-C with CVD incidence. (B) LDL-C with CVD mortality. (C) HDL-C with CVD incidence. (D) HDL-C with CVD mortality. Model adjusted for sex, age, race, alcohol consumption, smoking status, hypertension, diabetes, and BMI. Abbreviations as in Figure 1.

A significant U-shaped association was observed between LDL-C and incident CVD risk (Figure 3A). The inflection point of the curve occurred at 3.861 mmol/L. Below this threshold, CVD incidence risk decreased rapidly with increasing LDL-C concentrations (*P* nonlinear <0.001). Conversely, above 3.861 mmol/L, risk increased progressively with higher LDL-C levels.

**Figure 4:**
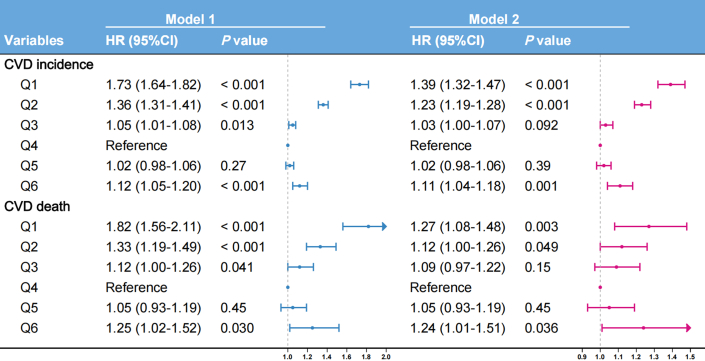
**Associations of Low-Density Lipoprotein Cholesterol Level With Cardiovascular Disease Risk** LDL-C level Q1: 0.276 to 2.211, Q2: 2.211 to 3.000, Q3: 3.000 to 3.600, Q4: 3.600 to 4.204, Q5: 4.204 to 5.145, Q6: 5.145 to 9.610. Model 1 adjusted for sex and age; model 2 additionally adjusted for race, alcohol consumption, smoking status, hypertension, diabetes, and BMI. Abbreviation as in Figure 1.

Similarly, LDL-C demonstrated a U-shaped relationship with CVD mortality risk (Figure 3B), with an inflection point at 3.792 mmol/L (*P* nonlinear = 0.001). Based on identified inflection points, we established the LDL-C Goldilocks zone (Q4: 3.600-4.204 mmol/L) as a provisional reference benchmark. Adjusted multivariable Cox regression analyses using the Goldilocks zone revealed significantly elevated risks: The lowest LDL-C group (Q1: 0.276-2.211 mmol/L) exhibited a 39% higher incident CVD risk (HR: 1.39; 95% CI: 1.32-1.47) and 27% higher CVD mortality risk (HR: 1.27; 95% CI: 1.08-1.48). The highest LDL-C group (Q6: 5.145-9.610 mmol/L) showed a 11% elevated incident CVD risk (HR: 1.11; 95% CI: 1.04-1.18) and 24% increase in CVD mortality (HR: 1.24; 95% CI: 1.01-1.51) (Figure 4).

### Association between HDL-C and CVD outcomes

HDL-C concentrations exhibited an L-shaped relationship with incident CVD risk (Figure 3C). The curve inflection occurred at 1.439 mmol/L, with incident CVD risk spiking sharply at concentrations below this threshold (*P* nonlinear <0.001). Above 1.439 mmol/L, risk demonstrated an attenuated reduction with increasing HDL-C levels.

**Figure 5:**
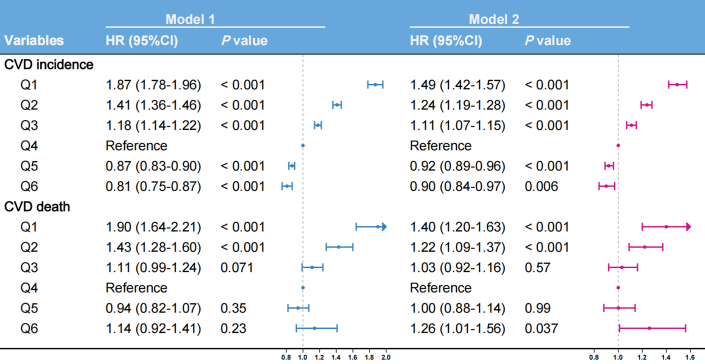
**Associations of High-Density Lipoprotein Cholesterol level with Cardiovascular Disease Risk** HDL-C level Q1: 0.252 to 0.935, Q2: 0.935 to 1.191, Q3: 1.191 to 1.421, Q4: 1.421 to 1.699, Q5: 1.699 to 2.174, Q6: 2.174 to 4.107. Model 1 adjusted for sex and age; model 2 additionally adjusted for race, alcohol consumption, smoking status, hypertension, diabetes, and BMI. Abbreviation as in Figure 1.

Conversely, HDL-C showed a U-shaped association with CVD mortality (Figure 3D), with inflection at 1.580 mmol/L (*P* nonlinear <0.001). Based on identified inflection points, we established the HDL-C Goldilocks zone (Q4: 1.421-1.699 mmol/L) as a provisional reference benchmark. Using the Goldilocks zone as the reference in adjusted multivariable Cox regression models, the lowest quintile (Q1: 0.252-0.935 mmol/L) had 40% greater CVD mortality (HR: 1.40; 95% CI: 1.20-1.63) and 49% higher incident CVD risk (HR: 1.49; 95% CI: 1.42-1.57). The highest quintile (Q5: 2.174-4.107 mmol/L) showed a 26% elevated CVD mortality risk (HR: 1.26; 95% CI: 1.01-1.56) but 9.8% reduced incident CVD risk (HR: 0.90; 95% CI: 0.84-0.97) (Figure 5).

### Risk stratification by combined LDL-C and HDL-C

Adjusted multivariable Cox regression models demonstrated significantly elevated cardiovascular risks in the high-risk stratum compared to the reference threshold group: a 55% greater incident CVD risk (HR: 1.55; 95% CI: 1.44-1.66) and 37% higher CVD mortality risk (HR: 1.37; 95% CI: 1.10-1.71). A significant linear dose-response relationship emerged across the incremental risk strata for both CVD mortality and incidence (*P* for trend <0.001). Per 1-category increase in risk stratification: CVD mortality risk rose by 9% (HR: 1.09; 95% CI: 1.05-1.13). Incident CVD risk increased by 10% (HR: 1.10; 95% CI: 1.09-1.12) (Figure 2). Based on the World Health Organization (WHO) European Region's 2024 CVD data report, we estimate that even with a 10% adjustment efficiency, optimizing LDL-C, HDL-C, and risk stratification to appropriate thresholds could reduce CVD-related deaths in the European Region by nearly 400,000 (Supplemental Figure 4).

### Subgroup and sensitivity analyses

**Figure 6:**
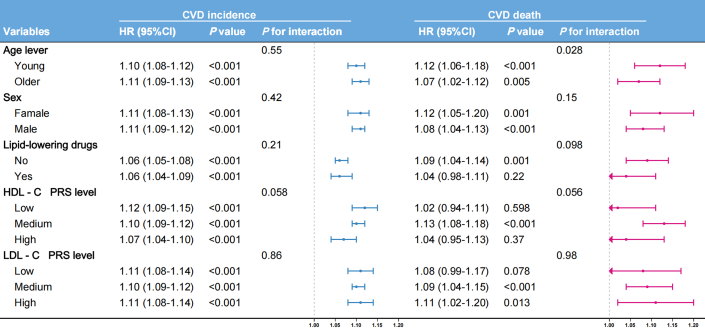
**Subgroup and Sensitivity Analyses** Model adjusted for sex, age, race, alcohol consumption, smoking status, hypertension, diabetes, and BMI. PRS = polygenic risk score; other abbreviations as in Figure 1.

Subgroup analyses of the association between the increasing risk strata and CVD outcomes are presented in Figure 6. Significant interaction was observed between age stratification and risk categories for CVD mortality (*P* for interaction = 0.028). For each incremental risk category, CVD mortality risk increased by 12% in the younger-older group (HR: 1.12; 95% CI: 1.06-1.18), significantly higher than the 7% increase in the older-elderly group (HR: 1.07; 95% CI: 1.02-1.12). No material difference by age stratification was observed for incident CVD risk. For both outcomes, no significant interactions were detected for sex, lipid-lowering medication use, or genetic predisposition to HDL-C and LDL-C levels (all *P* > 0.05). Landmark analysis excluding participants who died within 1, 3, and 5 years demonstrated consistent results (Supplemental Figures 5 to 7). The analysis results of the Fine-Gray subdistribution hazards models are basically consistent with the trend of the results of the main Cox model. (Supplemental Table 4).

## Discussion

This UKB analysis systematically demonstrates nonlinear associations (U/L-shaped curves) between LDL-C/HDL-C levels and CVD risk in older adults, revealing paradoxical risks associated with extremely low LDL-C and high HDL-C levels. Based on these observational associations, we preliminarily propose potential optimal ranges for dual lipid management (LDL-C: 3.600-4.204 mmol/L; HDL-C: 1.421-1.699 mmol/L). Moreover, this joint risk quantification only suggests the potential value of transitioning from univariable linear models to multivariable nonlinear frameworks, rather than establishing a new treatment paradigm, but still provides validated evidence for multimetric thresholds in geriatric lipid management.

### LDL-C

Our investigation corroborates prior research in confirming a U-shaped relationship between LDL-C level concentrations and CVD risk, revealing a noteworthy paradox: substantially low LDL-C levels exhibit a significant correlation with elevated CVD risk.^14^, ^15^, ^16^ In practice, doubling the statin dose achieves disproportionately limited efficacy by reducing LDL-C by only 6% (rule of 6), whereas adverse events increase disproportionately with escalating doses.^17^^,^^18^ However, The SPARCL (Stroke Prevention by Aggressive Reduction in Cholesterol Levels) trial first reported an association between low LDL-C levels and an increased incidence of HS.^19^ The REALITY study demonstrated that although only 3% of patients with ASCVD achieved the European Atherosclerosis Society/European Society of Cardiology guideline target of LDL-C <1.4 mmol/L (55 mg/dL) despite high-intensity lipid-lowering therapy, 25% experienced recurrent ASCVD events during follow-up.^20^ This highlights the limitations of traditional linear lipid management methods that focus solely on lowering LDL-C levels.

Distinct inflection points emerged for incident CVD (3.861 mmol/L) vs CVD mortality (3.792 mmol/L), possibly reflecting differential cumulative exposure windows required for atherogenesis compared to acute plaque rupture. Geriatric CVD progression involves decades-long synergism between modifiable (such as hypertension and diabetes) and nonmodifiable (such as genetic and epigenetic) risk factors.^21^ Conversely, lethal events frequently result from sudden plaque destabilization triggered by systemic inflammation, frailty exacerbation, or physiological stressors—processes potentially mitigated by prompt lipid-lowering therapy.^17^ Patients who develop CVD at higher LDL-C levels (>3.792 mmol/L) typically receive intensified statin regimens earlier, paradoxically reducing postdiagnosis mortality risk.^22^

Although elevated LDL-C concentrations (>3.792 mmol/L) are consistently associated with an increased CVD risk, the inverse relationship at lower concentrations (<3.792 mmol/L) is likely attributable to underlying comorbidities rather than a cardioprotective status. Extremely low LDL-C levels may signal malnutrition, chronic inflammation, or malignancy/liver disease-induced lipid dysregulation.^23^^,^^24^ Furthermore, hypocholesterolemia has been reported to increase susceptibility to infections and HSs—outcomes particularly consequential in older adults with compromised immunity.^19^^,^^25^, ^26^, ^27^ These factors may all lead to reverse causality bias, rendering the observed association between "extremely low LDL-C and high risk" in this study unsuitable for direct causal interpretation, yet they establish a foundation for future research to explore the underlying mechanisms driving this paradox.

### HDL-C

HDL-C is a well-integrated biomarker of cardiometabolic health. Despite extensive preclinical evidence supporting HDL-C's cardioprotective role, pharmacological interventions aimed at raising HDL-C levels have demonstrated disappointing clinical outcomes in multiple randomized controlled trials.^28^, ^29^, ^30^ The neutral outcomes suggesting potential oversight of threshold effects and nonlinear relationships underlying HDL-C—CVD risk associations. Notably, Madsen et al.^31^ reported U-shaped correlations, whereas Liu et al.^32^ identified U-curve relationships between HDL-C and subclinical atherosclerosis, collectively reinforcing our findings of distinct nonlinear associations in older adults—specifically, U-shaped for CVD mortality and L-shaped for incident CVD.

A consolidated scientific rationale for HDL-C's paradoxical effects: The heterogeneity in HDL-C dose-response relationships with CVD mortality and incidence suggests that beyond optimal levels, elevated HDL-C may reflect dysregulated lipid metabolism or chronic inflammation activation, thereby losing its monofunctional protective role.^33^^,^^34^ The negative slope of the U-shaped curve aligns with established cardioprotective mechanisms at moderate concentrations, whereas the positive slope at higher levels potentially arises from several factors: 1) structural alterations in oversized HDL particles that impede arterial cholesterol clearance;^35^ 2) functional impairment, where inflammation and oxidative stress diminish cholesterol efflux capacity, reversing antiatherogenic effects;^33^^,^^34^ 3) cytotoxic effects through suppression of endothelial progenitor cells, accelerating vascular aging;^36^ and 4) genetic mediation via variants that elevate HDL-C yet confer disease susceptibility.^37^ Collectively, these mechanisms delineate the pathophysiological basis for the paradoxical risks associated with very high HDL-C levels.

### Joint effect and guideline implications

Given the uncertainty of guidelines and consensus on the target indicators and scope of blood lipid management in the old population, the primary and secondary prevention of cardiovascular burden attributed to blood lipids in the context of aging cannot continue to be ignored. We focus on the primary control targets LDL-C of guidelines and HDL-C negatively correlated with CVD. The results indicate that LDL-C and HDL-C have optimal ranges for cardiovascular events and jointly provide important information for old cardiovascular prevention, whereas LDL-C alone cannot capture this information (Central Illustration).

**Central Illustration:**
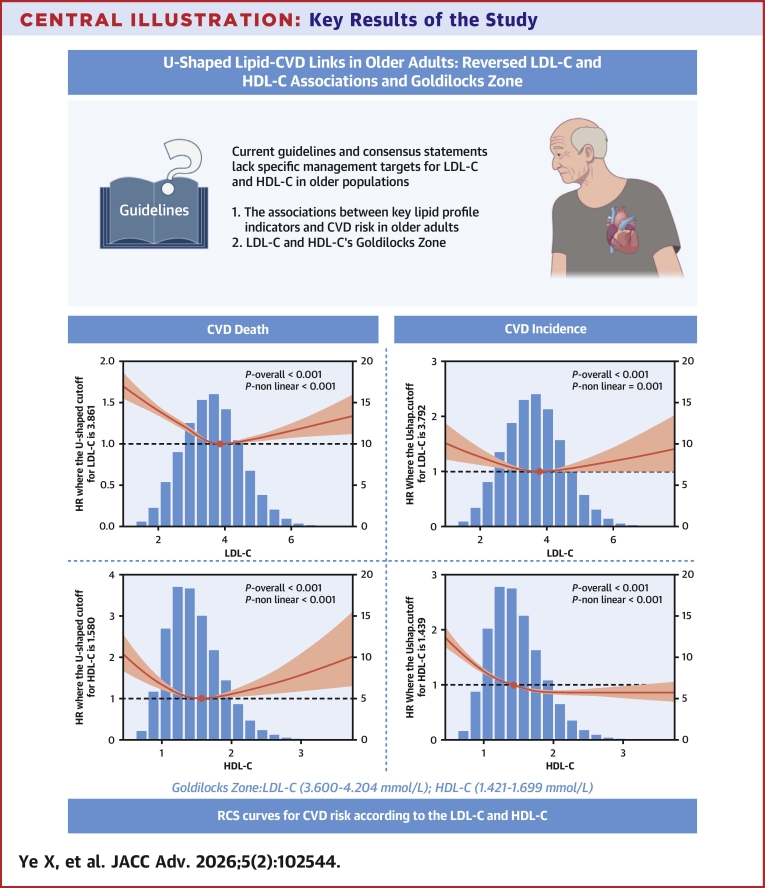

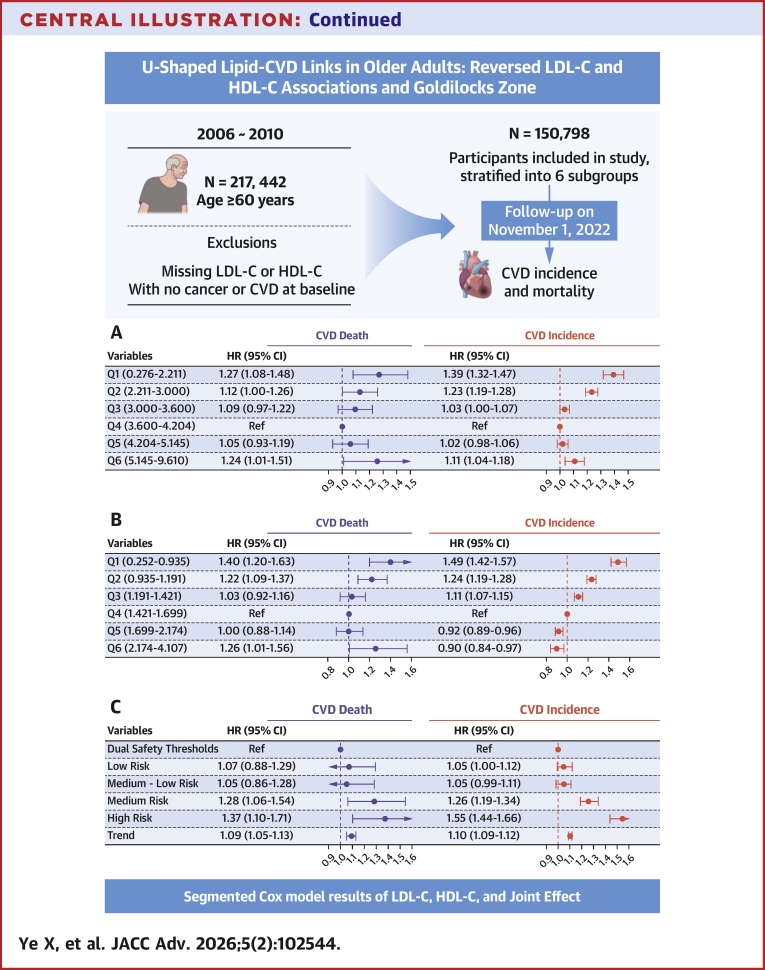
**Key Results of the Study** (A) LDL-C with CVD risk. (B) HDL-C with CVD risk. (C) Combined lipid profile with CVD risk. Model adjusted for sex, age, race, alcohol consumption, smoking status, hypertension, diabetes, and BMI. CVD = cardiovascular disease; HDL-C = high-density lipoprotein cholesterol; LDL-C = low-density lipoprotein cholesterol; RCS = restricted cubic spline.

This large-scale prospective cohort study demonstrates, for the first time, that simultaneous deviations of both LDL-C and HDL-C from their respective optimal ranges (LDL-C: 3.600-4.204 mmol/L; HDL-C: 1.421-1.699 mmol/L) exert superadditive effects on CVD risk (*P* < 0.001). This finding challenges the current single-metric-centric paradigm for lipid management and suggests the need to develop risk assessment algorithms that integrate both lipid metrics.

The divergence of our results from evidence derived in younger populations highlights the limitations of universal “lower-is-better” strategies in geriatric care. This compels the incorporation of age-stratified risk algorithms in future guidelines. Notably, the identified safety window reflects the critical importance of maintaining cholesterol homeostasis, wherein superadditive risk likely results from the converging dual U-shaped risk curves of LDL-C and HDL-C in relation to CVD outcomes. However, this association still requires validation through causal inference studies and should not be directly used as evidence for guideline revisions.

Current guideline variations in LDL-C targets for older adults inadequately address the hazards of extremely low LDL-C concentrations.^3^^,^^4^ Our analysis demonstrates that lowering LDL-C below 3.792 mmol/L increases CVD mortality risk—a threshold substantially higher than that recommended for very high-risk individuals (such as <1.8 mmol/L). This finding only provides hypothesis-generating evidence for "lower is not always better" in lipid management for the elderly population, rather than establishing clinically appropriate ranges. It also refines existing recommendations, supporting the relaxation of LDL-C targets in frail older populations while advocating for personalized, risk-based approaches.

The nonlinear relationship between HDL-C-CVD risk likewise highlights the complexity of lipid management in older adults, necessitating strategic equilibrium between atherosclerotic risk reduction and the avoidance of metabolic derangements. Importantly, although mean baseline HDL-C concentrations in major lipid intervention trials^28^, ^29^, ^30^ fell below our identified safety thresholds, substantial participant subsets likely exhibited increased HDL-C levels—a critical limitation, as these trials' generally omitted HDL-C stratification and predominantly focused on secondary prevention in coronary/diabetic cohorts. Our population-based study demonstrates that indiscriminately increasing HDL-C is insufficient to improve outcomes, indicating the need to integrate quantitative functional metrics (such as cholesterol efflux capacity) for precise vascular protection assessment.

Subgroup analyses revealed substantially greater risk elevation in lipid-lowering medication-naive participants (HR: 1.09) compared to treated individuals (HR: 1.04), suggesting that pharmacotherapy may partially mitigate the pathological damage from extreme lipid levels. Nevertheless, the known rates of statin intolerance among patients with acute coronary syndrome (7.2% to 16.2%)^38^ necessitate large-scale randomized controlled trials to confirm the risk-benefit ratios in older populations. Critically, the age-risk gradient interaction observed in CVD mortality compels a paradigm shift from uniform, lifelong targets toward age-adaptive modulation with a threshold adjusted by a decade.

To investigate the core nonlinear associations between LDL-C/HDL-C levels and CVD risk in older adults, we systematically evaluated penalized splines, RCS, natural splines, and fractional polynomials through theoretical derivation and simulation validation. RCS emerged as the optimal methodology, offering superior inflection sensitivity, curve smoothness, and clinically interpretable thresholds.^13^ Baseline health status and age-specific accuracy were ensured through stringent exclusion criteria (such as pre-existing CVD or cancer) and subgroup restriction to participants ≥65 years. Multidimensional subgroup analyses consistently validated the generalizability and robustness of our findings, with invariant nonlinear patterns and safety windows persisted across age strata, genders, PRS extremes (highest or lowest quartiles), and lipid-lowering medication users. The absence of significant interaction terms (*P* > 0.05) reinforces the biological plausibility of these results and establishes model utility across genetically stratified and pharmacologically managed populations across diverse clinical scenarios.

### Study limitations

Despite the study’s strengths—including the enhanced robustness of RCSs and rigorous sensitivity analyses—several limitations should be addressed. First, as an observational cohort analysis, causal interpretation of LDL-C/HDL-C levels and CVD risk remains unattainable. Second, although optimal multivariable adjustments were implemented, the potential for residual confounding persists. Third, the healthy volunteer bias inherent to the UKB participants potentially attenuates actual risk estimates. Fourth, reliance on single-baseline lipid measurements cannot capture dynamic fluctuations influencing outcomes. Therefore, future studies incorporating repeated lipid assessment will be essential to validate the threshold stability.

## Conclusions

This study demonstrates that lipid-CVD risk associations in older adults follow complex nonlinear patterns, suggesting the need to reassess lipid treatment targets for this population. We quantify an optimal lipid management window—LDL-C within 3.600 to 4.204 mmol/L and HDL-C within 1.421 to 1.699 mmol/L—representing a distinct "Goldilocks zone" that minimizes cardiovascular risk in this population. These evidence-based thresholds deliver critical epidemiological validation to transcend prevailing guideline limitations characterized by adults-aged stratification deficits and isolated LDL-C-centric paradigms, providing population-level targets for precision geriatric cardiovascular care.

Perspectives **COMPETENCY IN MEDICAL KNOWLEDGE:** Our findings redefine lipid management paradigms for older adults by demonstrating nonlinear associations of both LDL-C and HDL-C with CVD outcomes. The U-shaped relationship of LDL-C with incident CVD and mortality indicates that both excessively low (<2.211 mmol/L) and high levels confer risk, challenging the current guideline focus on unilateral LDL-C reduction. Concurrently, the L-shaped association of HDL-C with incident CVD and its U-shaped mortality relationship reveal complex, outcome-specific effects. Critically, the joint risk stratification proves lipid parameters must be considered interactively rather than in isolation.

**TRANSLATIONAL OUTLOOK:** Future guidelines need incorporate age-specific optimal ranges (LDL-C: 3.600-4.204 mmol/L; HDL-C: 1.421-1.699 mmol/L) and acknowledge the hazard of extremely low LDL-C. Randomized trials should evaluate whether maintaining dual-target lipid control reduces CVD events in older adults, particularly addressing risks associated with intensive LDL-C-lowering monotherapy. Population-based studies are needed to validate the clinical utility of combined LDL-C/HDL-C risk tier models.

## Funding support and author disclosures

This study was supported by the 10.13039/501100005089Beijing Municipal Natural Science Foundation (7252181), the Capital Health Development Special Fund (2024-2G-5033), the Noncommunicable Chronic Diseases-National Science and Technology Major Project (2023ZD0500901), the 10.13039/501100012166National Key Research and Development Program of China (2022YFC2503605), and the 10.13039/100014717National Natural Science Foundation of China (82173589, 82173590). The authors have reported that they have no relationships relevant to the contents of this paper to disclose.
