# Supplementary material for: U-Shaped Lipid-CVD Links in Older Adults: Reversed LDL-C and HDL-C Associations and Goldilocks Zone
Source: JACC Adv. 2026 Jan 22;5(2):102544. doi: 10.1016/j.jacadv.2025.102544 (PMC12861008; doi:10.1016/j.jacadv.2025.102544)
Supplement: Supplemental Tables 1-9 and Supplemental Figures 1-8 [file mmc1.docx]

**Supplementary Materials**

**Table S1** Definitions of Covariates.

**Table S2** ICD Codes and Coding Methods for All Outcomes.

**Table S3** Missing Rates of Baseline Variables in the UKB

**Table S4** Subdistribution Hazard Ratios for the Associations Between LDL-C&HDL-C Levels and CVD Mortality (Fine-Gray Hazards Models).

**Table S5** Univariable Cox Proportional Hazards Regression Analysis for CVD incedence.

**Table S6** Univariable Cox Proportional Hazards Regression Analysis for CVD Mortality.

**Table S7** Baseline data stratified by LDL-C level.

**Table S8** Baseline data stratified by HDL-C level.

**Table S9** E-value for LDL-C and HDL-C Levels on CVD Outcomes

**Figure S1** Variable Importance Assessment Using the Boruta Random Forest Algorithm.

**Figure S2** Change in Effect Size (CIE) Plots for LDL-C and HDL-C Levels on CVD Outcomes.

**Figure S3** Spearman Correlation Matrix Heatmap of Demographic, Lipid, Metabolic, and Lifestyle Variables.

**Figure S4** Public Health Impact of Multifactor Lipid Intervention in European Adults.

**Figure S5** Associations of lipid profile with the risk of CVD incidence and mortality in Cox models with restricted cubic splines after adjustment (excluding participants who died within the first year).

**Figure S6** Associations of lipid profile with the risk of CVD incidence and mortality in Cox models with restricted cubic splines after adjustment (excluding participants who died within 3year).

**Figure S7** Associations of lipid profile with the risk of CVD incidence and mortality in Cox models with restricted cubic splines after adjustment (excluding participants who died within 5 year).

**Figure S8** Log-Log Survival Curves (LLS): Proportional Hazards Assumption for LDL-C, HDL-C and CVD Outcomes.

**Table S1 Covariate definitions**

| Variable | Description | UK Biobank field ID |
| --- | --- | --- |
| Age | Age at recruitment | 21022 |
| Sex | Sex | 31 |
| Body mass index | Body mass index (BMI) | 21001 |
| Education | Qualifications | 6138 |
| Family history of CVD | Illnesses of father | 20107 |
|  | Illnesses of mother | 20110 |
| Smoking status | Smoking status | 20116 |
| Drinking status | Alcohol drinker status | 20117 |
| Hypertension | Vascular/heart problems diagnosed by doctor | 6150 |
|  | Age high blood pressure diagnosed | 2966 |
|  | Medication for cholesterol, blood pressure, diabetes, or take exogenous hormones | 6177,6153 |
|  | Diastolic blood pressure | 94,4079 |
|  | Systolic blood pressure | 93,4080 |
| Diabetes mellitus | [Diabetes diagnosed by doctor](https://biobank.ndph.ox.ac.uk/showcase/field.cgi?id=2443) | 2443 |
|  | Age diabetes diagnosed | 2976 |
|  | Medication for cholesterol, blood pressure, diabetes, or take exogenous hormones | 6177,6153 |
|  | Glycated haemoglobin (HbA1c) | 30750 |
|  | Glucose | 30740 |
| Activity group | IPAQ activity group | 22032 |
| Lipid-lowering medication use | Medication for cholesterol, blood pressure, diabetes, or take exogenous hormones | 6177,6153 |
| Cancer | ICD10 code (C00-C97, exclusions C44) | 41202, 41204, 40001, 40002 |
|  | Date of first in-patient diagnosis - main ICD10 | 41262 |

**Table S2 Coding methods and ICD code for all outcomes**

| Classification | Subtype | ICD10 code |
| --- | --- | --- |
| CVD |  | I60-I64, I20-I25, I42.0, I42.6, I42.7, I42.9, I50.0, I50.1, I50.9 |
|  | IHD | I20-I24, I25 |
|  | IS | I63 |
|  | HS | I60-I62 |
| CVD death |  | I60-I64, I20-I25, I42.0, I42.6, I42.7, I42.9, I50.0, I50.1, I50.9 |

IHD: ischemic heart disease; IS: ischemic stroke; HS: hemorrhagic stroke; ICD-10: international classification of diseases, 10th revision

**Table S3 Missing Rates of Baseline Variables in the UKB**

| Variables | Missing rate |
| --- | --- |
| Age, years | 0.000 |
| Body mass index, kg/m² | 0.004 |
| Blood glucose, mmol/L | 0.001 |
| C-reactive protein, mg/L | 0.002 |
| Diastolic blood pressure | 0.001 |
| Systolic blood pressure | 0.001 |
| High-density lipoprotein cholesterol, mmol/L | 0.000 |
| Glycated hemoglobin, mmol/mol | 0.051 |
| Low-density lipoprotein cholesterol, mmol/L | 0.000 |
| Low-density lipoprotein cholesterol polygenic risk score, no unit | 0.013 |
| High-density lipoprotein cholesterol polygenic risk score, no unit | 0.013 |
| Total cholesterol, mmol/L | 0.000 |
| Triglycerides, mmol/L | 0.001 |
| Waist circumference, cm | 0.002 |
| Race | 0.000 |
| Sex | 0.000 |
| Albumin, g/L | 0.001 |
| Educational level | 0.001 |
| Townsend Deprivation Index lever | 0.001 |
| International Physical Activity Questionnaire level | 0.210 |
| Smoker | 0.006 |
| Drinker | 0.002 |
| Lipid lowering drugs | 0.000 |
| Family history of cardiovascular disease | 0.000 |
| Diabetes | 0.000 |
| Hypertension | 0.000 |
| Lipoprotein(a) | 0.201 |

**Table S4** Subdistribution Hazard Ratios for the Associations Between LDL-C&HDL-C Levels and CVD Mortality (Fine-Gray Hazards Models).

| Variables | HR (95%CI) | *P* |
| --- | --- | --- |
| Low-density lipoprotein cholesterol level |  |  |
| Q1 | 1.21 (1.04-1.42) | 0.016 |
| Q2 | 1.09 (0.97-1.23) | 0.13 |
| Q3 | 1.07 (0.96-1.20) | 0.25 |
| Q4 | Reference | <0.001 |
| Q5 | 1.08 (0.95-1.22) | 0.24 |
| Q6 | 1.29 (1.06-1.57) | 0.013 |
| High-density lipoprotein cholesterol level |  |  |
| Q1 | 1.29 (1.10-1.50) | 0.002 |
| Q2 | 1.18 (1.05-1.32) | 0.006 |
| Q3 | 1.02 (0.91-1.14) | 0.75 |
| Q4 | Reference | <0.001 |
| Q5 | 1.01 (0.88-1.16) | 0.87 |
| Q6 | 1.29 (1.04-1.60) | 0.020 |

Low-density lipoprotein cholesterol level Q1: 0.276-2.211, Q2: 2.211-3.000, Q3: 3.000-3.600, Q4: 3.600-4.204, Q5: 4.204-5.145, Q6: 5.145-9.610. High-density lipoprotein cholesterol level Q1: 0.252-0.935, Q2: 0.935-1.191, Q3: 1.191-1.421, Q4: 1.421-1.699, Q5: 1.699-2.174, Q6: 2.174-4.107.

**Table S5 Univariable Cox Proportional Hazards Regression Analysis for CVD incedence**

| Variables | HR (95%CI) | *P* |
| --- | --- | --- |
| Race |  |  |
| White | Reference |  |
| Other | 0.95 (0.91-1) | 0.038 |
| Sex |  |  |
| Female | Reference |  |
| Male | 1.84 (1.79-1.88) | <0.001 |
| Educational level |  |  |
| Below high school | Reference |  |
| High school | 0.77 (0.74-0.81) | <0.001 |
| Post-secondary education | 0.76 (0.74-0.78) | <0.001 |
| Townsend Deprivation Index lever |  |  |
| Low | Reference |  |
| Low - Medium | 1.09 (1.05-1.12) | <0.001 |
| Medium | 1.14 (1.10-1.18) | <0.001 |
| Medium - High | 1.30 (1.25-1.36) | <0.001 |
| High | 1.54 (1.47-1.61) | <0.001 |
| International Physical Activity Questionnaire level |  |  |
| Low | Reference |  |
| Medium | 0.81 (0.78-0.84) | <0.001 |
| High | 0.82 (0.79-0.85) | <0.001 |
| Smoking |  |  |
| No | Reference |  |
| YES | 1.40 (1.37-1.44) | <0.001 |
| Drinking |  |  |
| No | Reference |  |
| YES | 0.87 (0.83-0.92) | <0.001 |
| Body mass index level |  |  |
| Emaciated | Reference |  |
| Underweight | 0.90 (0.73-1.11) | 0.33 |
| Normal weight | 1.26 (1.03-1.55) | 0.028 |
| Obese | 1.73 (1.41-2.13) | <0.001 |
| Diabetes |  |  |
| No | Reference |  |
| YES | 1.90 (1.84-1.97) | <0.001 |
| Hypertension |  |  |
| No | Reference |  |
| YES | 1.69 (1.64-1.74) | <0.001 |
| Lipid lowering drugs |  |  |
| No | Reference |  |
| YES | 1.93 (1.88-1.98) | <0.001 |
| Family history CVD |  |  |
| No | Reference |  |
| YES | 1.16 (1.13-1.18) | <0.001 |
| Low-density lipoprotein cholesterol level |  |  |
| Q1 | Reference |  |
| Q2 | 0.72 (0.69-0.76) | <0.001 |
| Q3 | 0.52 (0.50-0.55) | <0.001 |
| Q4 | 0.48 (0.46-0.50) | <0.001 |
| Q5 | 0.47 (0.45-0.49) | <0.001 |
| Q6 | 0.48 (0.45-0.52) | <0.001 |
| High-density lipoprotein cholesterol level |  |  |
| Q1 | Reference |  |
| Q2 | 0.71 (0.68-0.74) | <0.001 |
| Q3 | 0.55 (0.53-0.58) | <0.001 |
| Q4 | 0.43 (0.41-0.46) | <0.001 |
| Q5 | 0.35 (0.33-0.37) | <0.001 |
| Q6 | 0.31 (0.29-0.34) | <0.001 |
| Age | 1.09 (1.09-1.1) | <0.001 |
| Systolic blood pressure | 1.01 (1.01-1.01) | <0.001 |
| Diastolic blood pressure | 1.00 (1.00-1.01) | <0.001 |
| Total cholesterol | 0.81 (0.80-0.81) | <0.001 |
| Triglycerides | 1.13 (1.12-1.14) | <0.001 |
| Low-density lipoprotein cholesterol | 0.80 (0.78-0.81) | <0.001 |
| High-density lipoprotein cholesterol | 0.43 (0.41-0.44) | <0.001 |
| C-reactive protein | 1.02 (1.02-1.02) | <0.001 |
| Lipoprotein(a) | 1.00 (1.00-1.00) | <0.001 |
| Blood glucose | 1.09 (1.08-1.10) | <0.001 |
| Glycated hemoglobin A1c | 1.02 (1.02-1.02) | <0.001 |
| Waist circumference | 1.03 (1.03-1.03) | <0.001 |
| Body mass index | 1.05 (1.05-1.06) | <0.001 |
| Creatinine | 1.01 (1.00-1.01) | <0.001 |
| Uric acid | 1.00 (1.00-1.00) | <0.001 |
| Albumin | 0.96 (0.95-0.96) | <0.001 |
| Low-density lipoprotein cholesterol polygenic risk score | 0.95 (0.94-0.96) | <0.001 |
| High-density lipoprotein cholesterol polygenic risk score | 1.05 (1.03-1.06) | <0.001 |
| Townsend deprivation index | 1.04 (1.04-1.05) | <0.001 |

CI, confidence interval; CVD, cardiovascular disease. Low-density lipoprotein cholesterol level Q1: 0.276-2.211, Q2: 2.211-3.000, Q3: 3.000-3.600, Q4: 3.600-4.204, Q5: 4.204-5.145, Q6: 5.145-9.610. High-density lipoprotein cholesterol level Q1: 0.252-0.935, Q2: 0.935-1.191, Q3: 1.191-1.421, Q4: 1.421-1.699, Q5: 1.699-2.174, Q6: 2.174-4.107.

**Table S6 Univariable Cox Proportional Hazards Regression Analysis for CVD Mortality**

| Variables | HR (95%CI) | *P* |
| --- | --- | --- |
| Race |  |  |
| White | Reference |  |
| Other | 0.9 (0.78-1.04) | 0.14 |
| Sex |  |  |
| Female | Reference |  |
| Male | 2.38 (2.2-2.58) | <0.001 |
| Educational level |  |  |
| Below high school | Reference |  |
| High school | 0.79 (0.68-0.91) | 0.001 |
| Post-secondary education | 0.77 (0.70-0.84) | <0.001 |
| Townsend Deprivation Index lever |  |  |
| Low | Reference |  |
| Low - Medium | 1.15 (1.04-1.27) | 0.006 |
| Medium | 1.34 (1.19-1.51) | <0.001 |
| Medium - High | 1.75 (1.54-1.97) | <0.001 |
| High | 2.27 (2.00-2.58) | <0.001 |
| International Physical Activity Questionnaire level |  |  |
| Low | Reference |  |
| Medium | 0.72 (0.65-0.80) | <0.001 |
| High | 0.71 (0.64-0.79) | <0.001 |
| Smoking |  |  |
| No | Reference |  |
| YES | 1.76 (1.62-1.90) | <0.001 |
| Drinking |  |  |
| No | Reference |  |
| YES | 0.81 (0.69-0.96) | 0.013 |
| Body mass index level |  |  |
| Emaciated | Reference |  |
| Underweight | 0.50 (0.31-0.81) | 0.005 |
| Normal weight | 0.64 (0.39-1.03) | 0.064 |
| Obese | 0.89 (0.55-1.44) | 0.64 |
| Diabetes |  |  |
| No | Reference |  |
| YES | 2.59 (2.35-2.85) | <0.001 |
| Hypertension |  |  |
| No | Reference |  |
| YES | 2.09 (1.90-2.30) | <0.001 |
| Lipid lowering drugs |  |  |
| No | Reference |  |
| YES | 1.92 (1.77-2.07) | <0.001 |
| Family history CVD |  |  |
| No | Reference |  |
| YES | 1.03 (0.96-1.12) | 0.40 |
| Low-density lipoprotein cholesterol level |  |  |
| Q1 | Reference |  |
| Q2 | 0.65 (0.56-0.75) | <0.001 |
| Q3 | 0.50 (0.44-0.58) | <0.001 |
| Q4 | 0.42 (0.36-0.49) | <0.001 |
| Q5 | 0.41 (0.35-0.48) | <0.001 |
| Q6 | 0.44 (0.36-0.55) | <0.001 |
| High-density lipoprotein cholesterol level |  |  |
| Q1 | Reference |  |
| Q2 | 0.69 (0.60-0.79) | <0.001 |
| Q3 | 0.47 (0.41-0.55) | <0.001 |
| Q4 | 0.38 (0.33-0.44) | <0.001 |
| Q5 | 0.31 (0.27-0.37) | <0.001 |
| Q6 | 0.35 (0.28-0.44) | <0.001 |
| Age | 1.13 (1.12-1.15) | <0.001 |
| Systolic blood pressure | 1.01 (1.01-1.02) | <0.001 |
| Diastolic blood pressure | 1.01 (1.01-1.01) | <0.001 |
| Total cholesterol | 0.78 (0.76-0.81) | <0.001 |
| Triglycerides | 1.13 (1.09-1.17) | <0.001 |
| Low-density lipoprotein cholesterol | 0.76 (0.73-0.80) | <0.001 |
| High-density lipoprotein cholesterol | 0.40 (0.36-0.45) | <0.001 |
| C-reactive protein | 1.03 (1.03-1.04) | <0.001 |
| Lipoprotein(a) | 1.00 (1.00-1.00) | 0.008 |
| Blood glucose | 1.14 (1.12-1.16) | <0.001 |
| Glycated hemoglobin A1c | 1.03 (1.02-1.03) | <0.001 |
| Waist circumference | 1.03 (1.03-1.03) | <0.001 |
| Body mass index | 1.05 (1.04-1.06) | <0.001 |
| Creatinine | 1.01 (1.00-1.01) | <0.001 |
| Uric acid | 1.00 (1.00-1.00) | <0.001 |
| Albumin | 0.93 (0.91-0.94) | <0.001 |
| Low-density lipoprotein cholesterol polygenic risk score | 0.95 (0.92-0.99) | 0.010 |
| High-density lipoprotein cholesterol polygenic risk score | 1.02 (0.99-1.06) | 0.21 |
| Townsend deprivation index | 1.09 (1.08-1.10) | <0.001 |

CI, confidence interval; CVD, cardiovascular disease. Low-density lipoprotein cholesterol level Q1: 0.276-2.211, Q2: 2.211-3.000, Q3: 3.000-3.600, Q4: 3.600-4.204, Q5: 4.204-5.145, Q6: 5.145-9.610. High-density lipoprotein cholesterol level Q1: 0.252-0.935, Q2: 0.935-1.191, Q3: 1.191-1.421, Q4: 1.421-1.699, Q5: 1.699-2.174, Q6: 2.174-4.107.

**Table S7** **Baseline data stratified by LDL-C level**

| **LDL-C**  **mmol/L** | **Overall** | **Q1**  **0.276-2.211** | **Q2**  **2.211-3.000** | **Q3**  **3.000-3.600** | **Q4**  **3.600-4.204** | **Q5**  **4.204-5.145** | **Q6**  **5.145-9.610** |
| --- | --- | --- | --- | --- | --- | --- | --- |
| N | 150,798 | 7,550 | 30,152 | 37,752 | 37,672 | 30,142 | 7,530 |
| Age, years | 64.0 (62.0, 66.0) | 65.0 (62.0, 67.0) | 64.0 (62.0, 67.0) | 64.0 (62.0, 66.0) | 64.0 (61.0, 66.0) | 64.0 (61.0, 66.0) | 63.0 (61.0, 66.0) |
| Body mass index, kg/m² | 26.9 (24.5, 29.8) | 28.2 (25.4, 31.7) | 27.3 (24.6, 30.5) | 26.7 (24.2, 29.6) | 26.7 (24.3, 29.5) | 26.9 (24.6, 29.6) | 27.0 (24.8, 29.7) |
| Blood glucose, mmol/L | 5.0 (4.7, 5.4) | 5.2 (4.8, 6.1) | 5.1 (4.7, 5.6) | 5.0 (4.7, 5.4) | 5.0 (4.7, 5.3) | 5.0 (4.7, 5.3) | 5.0 (4.7, 5.4) |
| C-reactive protein, mg/L | 1.5 (0.8, 3.0) | 1.3 (0.7, 2.9) | 1.4 (0.7, 2.9) | 1.4 (0.7, 2.9) | 1.5 (0.8, 3.0) | 1.6 (0.9, 3.1) | 1.8 (0.9, 3.3) |
| Diastolic blood pressure, mmHg | 82.5 (76.0, 89.0) | 80.0 (74.0, 87.0) | 81.5 (75.0, 88.0) | 82.0 (75.5, 89.0) | 82.5 (76.5, 89.5) | 83.5 (77.0, 90.0) | 84.0 (77.5, 90.5) |
| Systolic blood pressure, mmHg | 143.0 (131.0, 155.5) | 141.0 (130.0, 153.0) | 142.5 (131.0, 155.0) | 142.5 (130.5, 155.0) | 143.0 (131.0, 156.0) | 143.5 (131.5, 157.0) | 145.0 (132.5, 158.0) |
| High-density lipoprotein cholesterol, mmol/L | 1.4 (1.2, 1.7) | 1.2 (1.0, 1.5) | 1.3 (1.1, 1.6) | 1.4 (1.2, 1.7) | 1.5 (1.2, 1.7) | 1.5 (1.3, 1.7) | 1.5 (1.3, 1.8) |
| Glycated hemoglobin, mmol/mol | 36.1 (33.9, 38.6) | 38.5 (35.1, 45.9) | 36.6 (34.1, 39.9) | 35.8 (33.5, 38.3) | 35.8 (33.6, 38.0) | 36.1 (34.0, 38.2) | 36.5 (34.4, 38.6) |
| Low-density lipoprotein cholesterol, mmol/L | 3.6 (3.0, 4.2) | 2.0 (1.8, 2.1) | 2.7 (2.5, 2.9) | 3.3 (3.2, 3.5) | 3.9 (3.7, 4.0) | 4.5 (4.4, 4.8) | 5.5 (5.3, 5.8) |
| Low-density lipoprotein cholesterol polygenic risk score, no unit | -0.06 (-0.75, 0.60) | -0.42 (-1.15, 0.29) | -0.23 (-0.98, 0.47) | -0.25 (-0.93, 0.42) | -0.07 (-0.70, 0.55) | 0.23 (-0.37, 0.82) | 0.56 (-0.02, 1.14) |
| High-density lipoprotein cholesterol polygenic risk score, no unit | 0.02 ± 1.01 | -0.02 ± 1.04 | 0.03 ± 1.02 | 0.06 ± 1.01 | 0.05 ± 1.01 | -0.01 ± 1.00 | -0.08 ± 0.99 |
| Total cholesterol, mmol/L | 5.8 (5.0, 6.6) | 3.6 (3.3, 3.9) | 4.6 (4.3, 4.9) | 5.4 (5.1, 5.7) | 6.1 (5.9, 6.4) | 7.0 (6.7, 7.3) | 8.2 (7.8, 8.6) |
| Triglycerides, mmol/L | 1.6 (1.1, 2.2) | 1.3 (0.9, 1.9) | 1.4 (1.0, 2.0) | 1.5 (1.1, 2.1) | 1.6 (1.2, 2.2) | 1.8 (1.3, 2.4) | 2.1 (1.5, 2.8) |
| Waist circumference, cm | 91.0 (82.0, 100.0) | 97.0 (88.0, 106.0) | 93.0 (84.0, 102.0) | 91.0 (81.0, 99.0) | 90.0 (81.0, 98.0) | 90.0 (82.0, 98.0) | 89.0 (82.0, 97.0) |
| White Race | 10,557 (7.0) | 618 (8.2) | 2,265 (7.5) | 2,767 (7.3) | 2,546 (6.8) | 1,927 (6.4) | 434 (5.8) |
| Male Sex | 70,643 (46.9) | 5,153 (68.3) | 17,215 (57.1) | 18,692 (49.5) | 16,529 (43.9) | 11,062 (36.7) | 1,992 (26.5) |
| Albumin, g/L | 44.9 (43.2, 46.5) | 44.6 (42.8, 46.5) | 44.7 (43.0, 46.4) | 44.7 (43.0, 46.4) | 44.8 (43.2, 46.5) | 45.1 (43.5, 46.8) | 45.6 (43.9, 47.3) |
| Educational level |  |  |  |  |  |  |  |
| Below high school | 97,210 (64.5) | 5,197 (68.8) | 19,835 (65.8) | 23,948 (63.4) | 23,825 (63.2) | 19,433 (64.5) | 4,972 (66.0) |
| High school | 13,577 (9.0) | 569 (7.5) | 2,563 (8.5) | 3,518 (9.3) | 3,413 (9.1) | 2,812 (9.3) | 702 (9.3) |
| Post-secondary education | 40,011 (26.5) | 1,784 (23.6) | 7,754 (25.7) | 10,286 (27.3) | 10,434 (27.7) | 7,897 (26.2) | 1,856 (24.7) |
| Townsend Deprivation Index lever |  |  |  |  |  |  |  |
| Low | 53,443 (35.4) | 2,305 (30.5) | 10,017 (33.2) | 13,453 (35.6) | 13,836 (36.7) | 11,096 (36.8) | 2,736 (36.3) |
| Low - Medium | 46,732 (31.0) | 2,225 (29.5) | 9,134 (30.3) | 11,697 (31.0) | 11,733 (31.2) | 9,542 (31.7) | 2,401 (31.9) |
| Medium | 23,607 (15.7) | 1,215 (16.1) | 4,863 (16.1) | 5,903 (15.6) | 5,854 (15.5) | 4,593 (15.2) | 1,179 (15.7) |
| Medium - High | 15,683 (10.4) | 940 (12.5) | 3,424 (11.4) | 3,928 (10.4) | 3,705 (9.8) | 2,944 (9.8) | 742 (9.9) |
| High | 11,333 (7.5) | 865 (11.5) | 2,714 (9.0) | 2,771 (7.3) | 2,544 (6.8) | 1,967 (6.5) | 472 (6.3) |
| International Physical Activity Questionnaire level |  |  |  |  |  |  |  |
| Low | 21,991 (14.6) | 1,431 (19.0) | 4,853 (16.1) | 5,302 (14.0) | 5,158 (13.7) | 4,216 (14.0) | 1,031 (13.7) |
| Medium | 65,070 (43.2) | 3,379 (44.8) | 12,811 (42.5) | 16,222 (43.0) | 16,242 (43.1) | 13,043 (43.3) | 3,373 (44.8) |
| High | 63,737 (42.3) | 2,740 (36.3) | 12,488 (41.4) | 16,228 (43.0) | 16,272 (43.2) | 12,883 (42.7) | 3,126 (41.5) |
| Current/Former smoker | 73,696 (48.9) | 4,172 (55.3) | 15,894 (52.7) | 18,385 (48.7) | 17,755 (47.1) | 14,053 (46.6) | 3,437 (45.6) |
| Current/Former drinker | 143,760 (95.3) | 7,134 (94.5) | 28,634 (95.0) | 36,056 (95.5) | 35,991 (95.5) | 28,759 (95.4) | 7,186 (95.4) |
| Lipid lowering drugs | 35,435 (23.5) | 5,923 (78.5) | 16,771 (55.6) | 8,232 (21.8) | 2,950 (7.8) | 1,249 (4.1) | 310 (4.1) |
| Family history of cardiovascular disease | 61,898 (41.1) | 3,105 (41.1) | 12,784 (42.4) | 15,298 (40.5) | 15,230 (40.4) | 12,316 (40.9) | 3,165 (42.0) |
| Diabetes | 12,922 (8.6) | 2,850 (37.8) | 4,864 (16.1) | 2,423 (6.4) | 1,515 (4.0) | 1,022 (3.4) | 248 (3.3) |
| Hypertension | 103,262 (68.5) | 6,030 (79.9) | 22,292 (73.9) | 25,331 (67.1) | 24,731 (65.7) | 19,828 (65.8) | 5,050 (67.1) |

Data are presented as median (25th–75th percentiles), mean ± standard deviation, or count (percentage, %). Comparisons across groups significant for all variables at p<0.001.

**Table S8 Baseline data stratified by HDL-C level**

| **Characteristics** | **Overall** | **Q1**  **0.252-0.935** | **Q2**  **0.935-1.191** | **Q3**  **1.191-1.421** | **Q4**  **1.421-1.699** | **Q5**  **1.699-**2.174 | **Q6**  **2.174-4.107** |
| --- | --- | --- | --- | --- | --- | --- | --- |
| N | 150798 | 7611 | 30136 | 37776 | 37602 | 30148 | 7525 |
| Age, years | 64.0 (62.0, 66.0) | 64.0 (62.0, 66.0) | 64.0 (62.0, 66.0) | 64.0 (62.0, 66.0) | 64.0 (62.0, 66.0) | 64.0 (61.0, 66.0) | 64.0 (62.0, 66.0) |
| Body mass index, kg/m² | 26.9 (24.5, 29.8) | 29.5 (27.0, 32.5) | 28.5 (26.1, 31.3) | 27.5 (25.2, 30.4) | 26.5 (24.2, 29.2) | 25.2 (23.1, 27.8) | 24.0 (22.0, 26.4) |
| Blood glucose, mmol/L | 5.0 (4.7, 5.4) | 5.1 (4.7, 5.9) | 5.0 (4.7, 5.5) | 5.0 (4.7, 5.4) | 5.0 (4.7, 5.4) | 5.0 (4.7, 5.4) | 5.0 (4.7, 5.4) |
| C-reactive protein, mg/L | 1.5 (0.8, 3.0) | 2.1 (1.0, 4.1) | 1.9 (1.0, 3.6) | 1.6 (0.9, 3.2) | 1.4 (0.7, 2.8) | 1.2 (0.6, 2.3) | 1.0 (0.5, 1.9) |
| Diastolic blood pressure, mmHg | 82.5 (76.0, 89.0) | 83.0 (76.5, 89.5) | 83.5 (77.0, 90.0) | 83.0 (76.5, 90.0) | 82.0 (75.5, 89.0) | 81.0 (75.0, 88.0) | 81.0 (74.0, 88.0) |
| Systolic blood pressure, mmHg | 143.0 (131.0, 155.5) | 143.0 (131.5, 155.0) | 143.0 (132.0, 155.5) | 143.5 (131.5, 156.0) | 142.5 (130.5, 155.5) | 142.0 (129.5, 155.5) | 143.5 (129.5, 157.5) |
| High-density lipoprotein cholesterol, mmol/L | 1.4 (1.2, 1.7) | 0.9 (0.8, 0.9) | 1.1 (1.0, 1.1) | 1.3 (1.3, 1.4) | 1.6 (1.5, 1.6) | 1.9 (1.8, 2.0) | 2.4 (2.3, 2.5) |
| Glycated hemoglobin, mmol/mol | 36.1 (33.9, 38.6) | 37.6 (34.5, 42.5) | 36.7 (34.2, 39.9) | 36.3 (34.0, 38.9) | 36.0 (33.8, 38.2) | 35.6 (33.6, 37.7) | 35.4 (33.4, 37.4) |
| Low-density lipoprotein cholesterol, mmol/L | 3.6 (3.0, 4.2) | 3.0 (2.4, 3.5) | 3.4 (2.8, 4.0) | 3.6 (3.0, 4.3) | 3.7 (3.1, 4.3) | 3.7 (3.2, 4.3) | 3.6 (3.1, 4.2) |
| Low-density lipoprotein cholesterol polygenic risk score, no unit | -0.06 (-0.75, 0.60) | -0.42 (-1.15, 0.29) | -0.23 (-0.98, 0.47) | -0.25 (-0.93, 0.42) | -0.07 (-0.70, 0.55) | 0.23 (-0.37, 0.82) | 0.56 (-0.02, 1.14) |
| High-density lipoprotein cholesterol polygenic risk score, no unit | 0.02 ± 1.01 | -0.02 ± 1.04 | 0.03 ± 1.02 | 0.06 ± 1.01 | 0.05 ± 1.01 | -0.01 ± 1.00 | -0.08 ± 0.99 |
| Total cholesterol, mmol/L | 5.8 (5.0, 6.6) | 4.6 (3.9, 5.3) | 5.2 (4.5, 6.0) | 5.7 (4.9, 6.4) | 5.9 (5.2, 6.7) | 6.2 (5.6, 6.9) | 6.6 (5.9, 7.3) |
| Triglycerides, mmol/L | 1.6 (1.1, 2.2) | 2.5 (1.8, 3.4) | 2.1 (1.5, 2.8) | 1.7 (1.3, 2.3) | 1.5 (1.1, 1.9) | 1.2 (0.9, 1.6) | 1.0 (0.8, 1.3) |
| Waist circumference, cm | 91.0 (82.0, 100.0) | 102.0 (95.0, 110.0) | 98.0 (91.0, 105.0) | 93.0 (86.0, 101.0) | 88.0 (81.0, 96.0) | 83.0 (76.0, 91.0) | 78.0 (72.0, 86.0) |
| White Race | 10,557 (7.0) | 577 (7.6) | 2,052 (6.8) | 2,552 (6.8) | 2,612 (7.0) | 2,211 (7.3) | 553 (7.4) |
| Male Sex | 70,643 (46.9) | 6,628 (87.1) | 21,728 (72.1) | 20,439 (54.1) | 13,955 (37.1) | 6,834 (22.7) | 1,059 (14.1) |
| Albumin, g/L | 44.9 (43.2, 46.5) | 44.4 (42.7, 46.1) | 44.6 (43.0, 46.3) | 44.7 (43.1, 46.4) | 44.9 (43.3, 46.5) | 45.1 (43.5, 46.8) | 45.7 (44.0, 47.5) |
| Educational level |  |  |  |  |  |  |  |
| Below high school | 97,210 (64.5) | 5,213 (68.5) | 20,312 (67.4) | 24,996 (66.2) | 24,077 (64.0) | 18,386 (61.0) | 4,226 (56.2) |
| High school | 13,577 (9.0) | 634 (8.3) | 2,481 (8.2) | 3,283 (8.7) | 3,368 (9.0) | 2,984 (9.9) | 827 (11.0) |
| Post-secondary education | 40,011 (26.5) | 1,764 (23.2) | 7,343 (24.4) | 9,497 (25.1) | 10,157 (27.0) | 8,778 (29.1) | 2,472 (32.9) |
| Townsend Deprivation Index lever |  |  |  |  |  |  |  |
| Low | 53,443 (35.4) | 2,389 (31.4) | 10,175 (33.8) | 13,350 (35.3) | 13,516 (35.9) | 11,235 (37.3) | 2,778 (36.9) |
| Low - Medium | 46,732 (31.0) | 2,301 (30.2) | 9,254 (30.7) | 11,693 (31.0) | 11,715 (31.2) | 9,462 (31.4) | 2,307 (30.7) |
| Medium | 23,607 (15.7) | 1,214 (16.0) | 4,799 (15.9) | 5,892 (15.6) | 5,914 (15.7) | 4,613 (15.3) | 1,175 (15.6) |
| Medium - High | 15,683 (10.4) | 900 (11.8) | 3,295 (10.9) | 3,932 (10.4) | 3,833 (10.2) | 2,972 (9.9) | 751 (10.0) |
| High | 11,333 (7.5) | 807 (10.6) | 2,613 (8.7) | 2,909 (7.7) | 2,624 (7.0) | 1,866 (6.2) | 514 (6.8) |
| International Physical Activity Questionnaire level |  |  |  |  |  |  |  |
| Low | 21,991 (14.6) | 1,799 (23.6) | 5,483 (18.2) | 5,798 (15.4) | 4,920 (13.1) | 3,285 (10.9) | 706 (9.4) |
| Medium | 65,070 (43.2) | 3,256 (42.8) | 13,218 (43.9) | 16,422 (43.5) | 16,272 (43.3) | 12,857 (42.7) | 3,045 (40.5) |
| High | 63,737 (42.3) | 2,556 (33.6) | 11,435 (37.9) | 15,556 (41.2) | 16,410 (43.6) | 14,006 (46.5) | 3,774 (50.2) |
| Current/Former smoker | 73,696 (48.9) | 4,432 (58.2) | 16,183 (53.7) | 18,620 (49.3) | 17,389 (46.2) | 13,568 (45.0) | 3,504 (46.6) |
| Current/Former drinker | 143,760 (95.3) | 7,138 (93.8) | 28,534 (94.7) | 35,939 (95.1) | 35,840 (95.3) | 28,974 (96.1) | 7,335 (97.5) |
| Lipid lowering drugs | 35,435 (23.5) | 3,139 (41.2) | 9,768 (32.4) | 9,662 (25.6) | 7,403 (19.7) | 4,468 (14.8) | 995 (13.2) |
| Family history of cardiovascular disease | 61,898 (41.1) | 2,949 (38.8) | 12,051 (40.0) | 15,467 (40.9) | 15,704 (41.8) | 12,705 (42.1) | 3,022 (40.2) |
| Diabetes | 12,922 (8.6) | 1,932 (25.4) | 4,242 (14.1) | 3,251 (8.6) | 2,106 (5.6) | 1,120 (3.7) | 271 (3.6) |
| Hypertension | 103,262 (68.5) | 5,900 (77.5) | 22,068 (73.2) | 26,641 (70.5) | 24,950 (66.4) | 18,947 (62.9) | 4,756 (63.2) |

Data are presented as median (25th–75th percentiles), mean ± standard deviation, or count (percentage, %). *Comparisons across groups were significant for all variables (p < 0.001) except for race (p = 0.007).*

**Table S9** E-value for LDL-C and HDL-C Levels on CVD Outcomes

| Variables | LDL-C Model 2 HR(95%CI) | E-value (lower 95%CI) | HDL-C Model 2 HR(95%CI) | E-value (CI) |
| --- | --- | --- | --- | --- |
| **CVD incidence** |  |  |  |  |
| Q1 | 1.39(1.32-1.47) | 1.823 (1.719) | 1.49(1.42-1.57) | 1.965 (1.866) |
| Q2 | 1.23(1.19-1.28) | 1.576 (1.508) | 1.24(1.19-1.28) | 1.593 (1.508) |
| Q3 | 1.03(1.00-1.07) |  | 1.11(1.07-1.15) | 1.359 (1.272) |
| Q4 | Reference |  | Reference |  |
| Q5 | 1.02(0.98-1.06) |  | 0.92(0.89-0.96) | 1.311 (1.201) |
| Q6 | 1.11(1.04-1.18) | 1.359 (1.196) | 0.90(0.84-0.97) | 1.361 (1.169) |
| **CVD death** |  |  |  |  |
| Q1 | 1.27(1.08-1.48) | 1.641 (1.295) | 1.40(1.20-1.63) | 1.838 (1.526) |
| Q2 | 1.12(1.00-1.26) |  | 1.22(1.09-1.37) | 1.559 (1.317) |
| Q3 | 1.09(0.97-1.22) |  | 1.03(0.92-1.16) |  |
| Q4 | Reference |  | Reference |  |
| Q5 | 1.05(0.93-1.19) |  | 1.00(0.88-1.14) |  |
| Q6 | 1.24(1.01-1.51) | 1.593 (1.090) | 1.26(1.01-1.56) | 1.625 (1.090) |

CI, confidence interval; HR, hazard ratio; LDL-C, low-density lipoprotein cholesterol; HDL-C, high-density lipoprotein cholesterol; CVD, cardiovascular disease. LDL-C level Q1: 0.276-2.211, Q2: 2.211-3.000, Q3: 3.000-3.600, Q4: 3.600-4.204, Q5: 4.204-5.145, Q6: 5.145-9.610. HDL-C level Q1: 0.252-0.935, Q2: 0.935-1.191, Q3: 1.191-1.421, Q4: 1.421-1.699, Q5: 1.699-2.174, Q6: 2.174-4.107.

* E-values represent the minimum strength of association (on the risk ratio scale) that an unmeasured confounder would need to have with both the exposure and outcome to fully explain away the observed association. E-value (lower 95%CI) indicates the E-value for the lower bound of the 95% confidence interval.

| 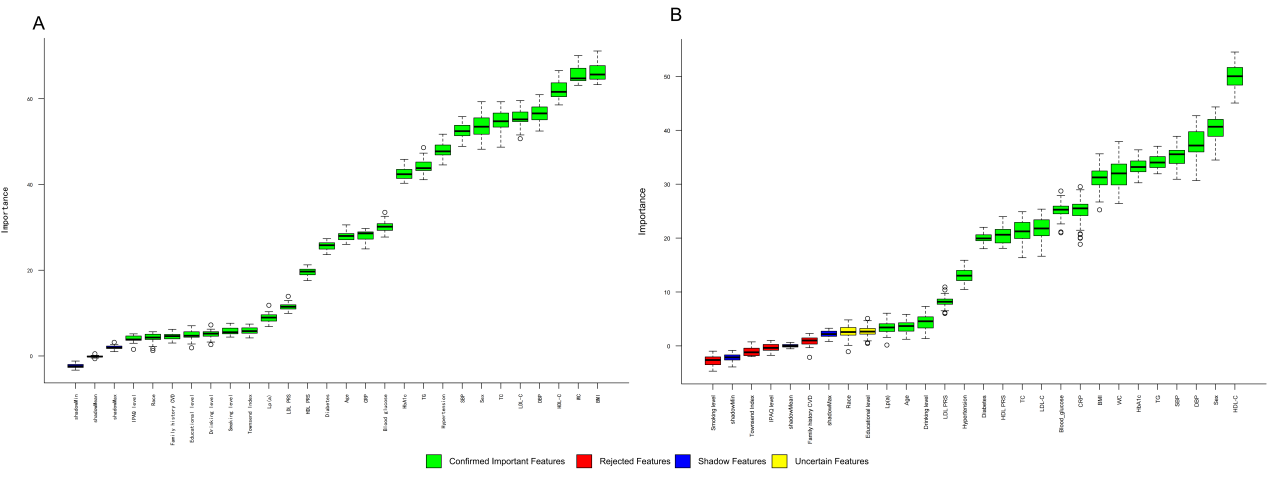 |
| --- |
| **Figure S1 Result of Boruta algorithm based on random forests to evaluate variable importance. A: CVD incidence’s variable importance. B: CVD mortality’s variable importance**  Ti: Townsend_Index; IPAQ: international physical activity questionnaire; SBP: systolic blood pressure; DBP: diastolic blood pressure; TC: total cholesterol; TG: triglyceride; LDL-C: low-density lipoprotein cholesterol; HDL-C: high-density lipoprotein cholesterol; CRP: C-Reactive Protein; Lp(a): apolipoprotein A; BMI: body mass index; WC: waist circumference; Alb: Albumin; PRS: polygenic risk score; HbA1c: Glycated Hemoglobin A1c; Cr: Creatinine; UA: uric acid. |

**
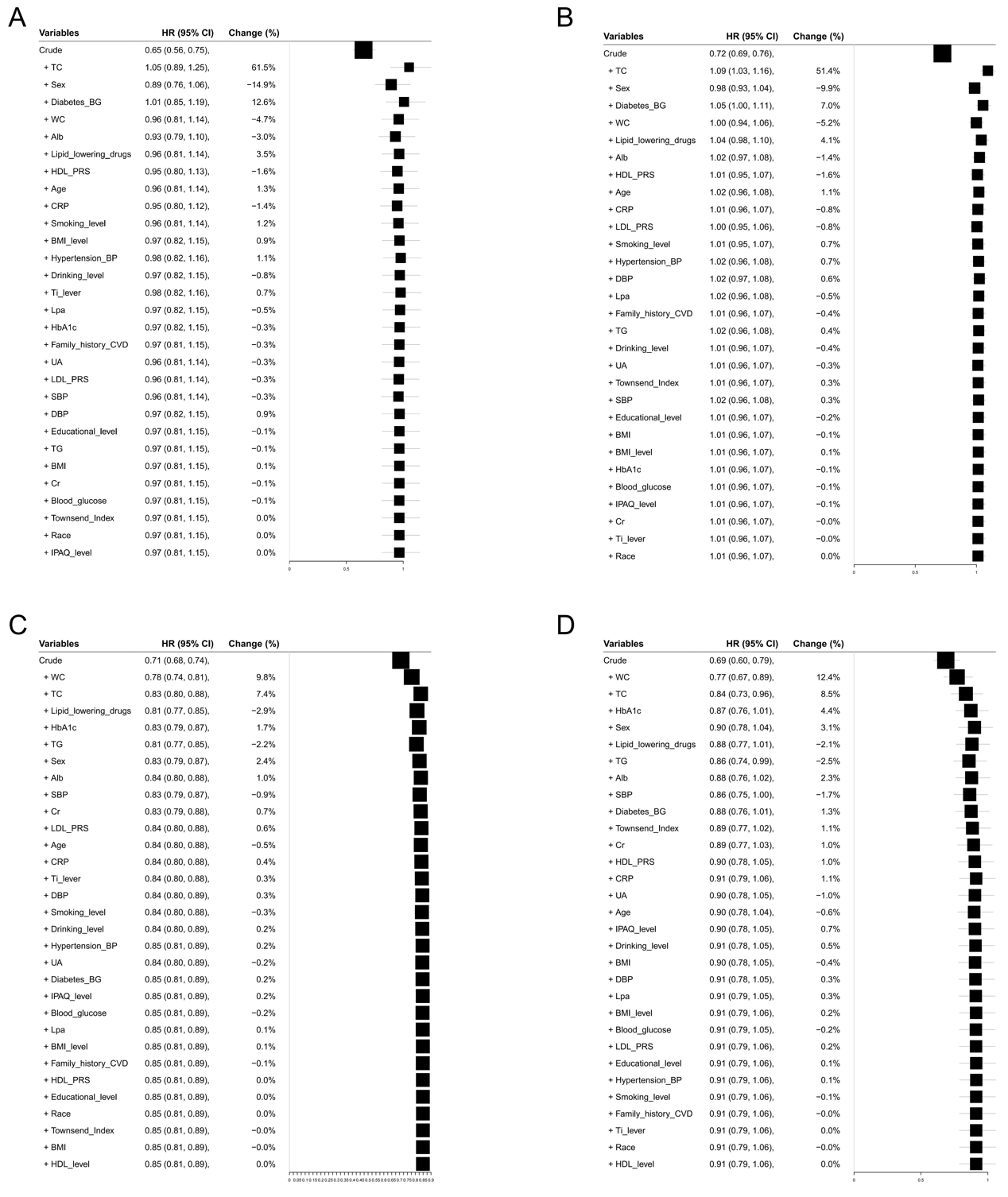
**

**Figure S2** Change in Effect Size (CIE) Plots for LDL-C and HDL-C Levels on CVD Outcomes.

A: LDL-C with CVD mortality. B: LDL-C with CVD incidence. C: HDL-C with CVD incidence. D: HDL-C with CVD mortality.


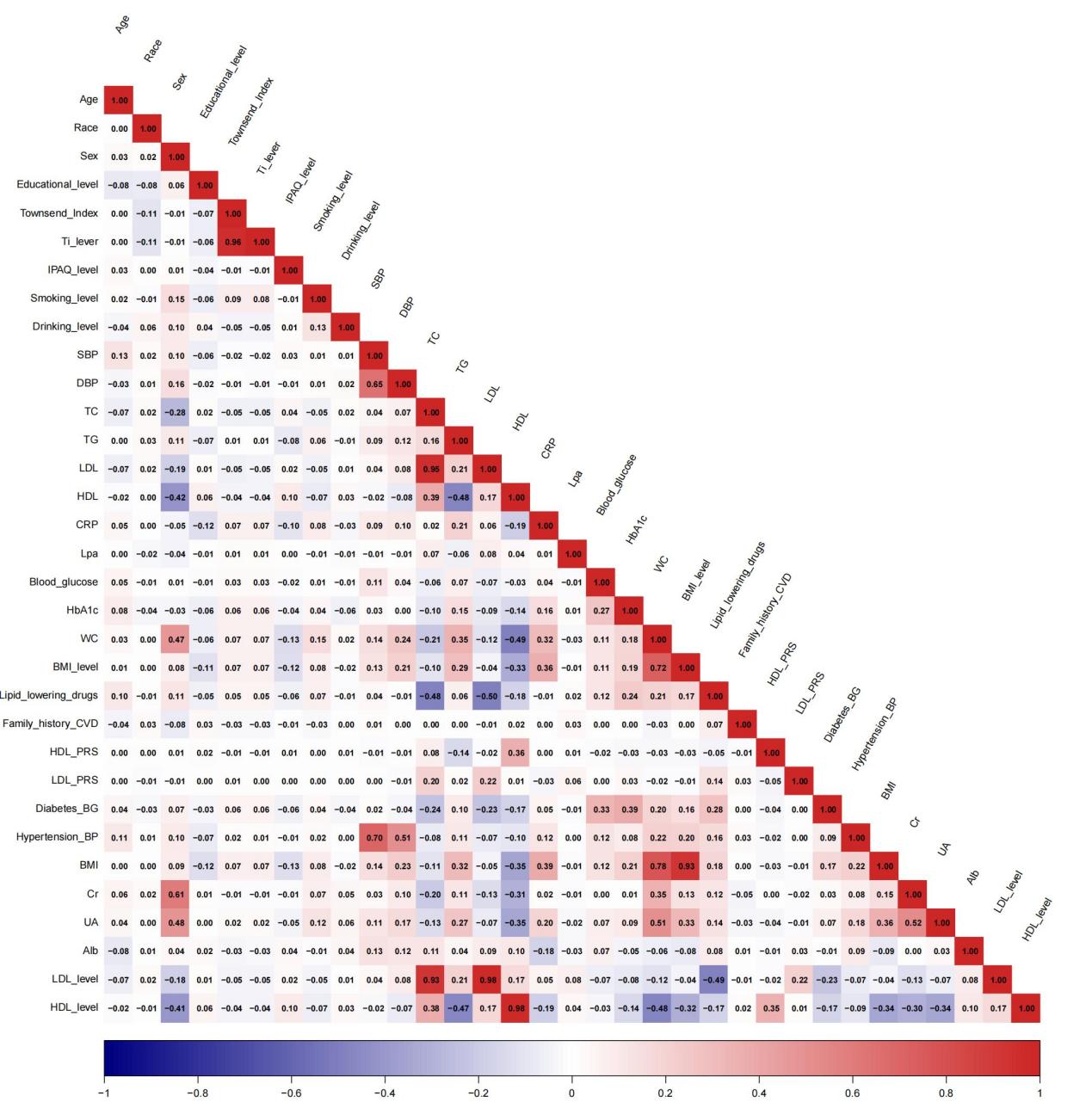


**Figure S3** Spearman Correlation Matrix Heatmap of Demographic, Lipid, Metabolic, and Lifestyle Variables.

Ti: Townsend_Index; IPAQ: international physical activity questionnaire; SBP: systolic blood pressure; DBP: diastolic blood pressure; TC: total cholesterol; TG: triglyceride; LDL-C: low-density lipoprotein cholesterol; HDL-C: high-density lipoprotein cholesterol; CRP: C-Reactive Protein; Lp(a): apolipoprotein A; BMI: body mass index; WC: waist circumference; Alb: Albumin; PRS: polygenic risk score; HbA1c: Glycated Hemoglobin A1c; Cr: Creatinine; UA: uric acid.


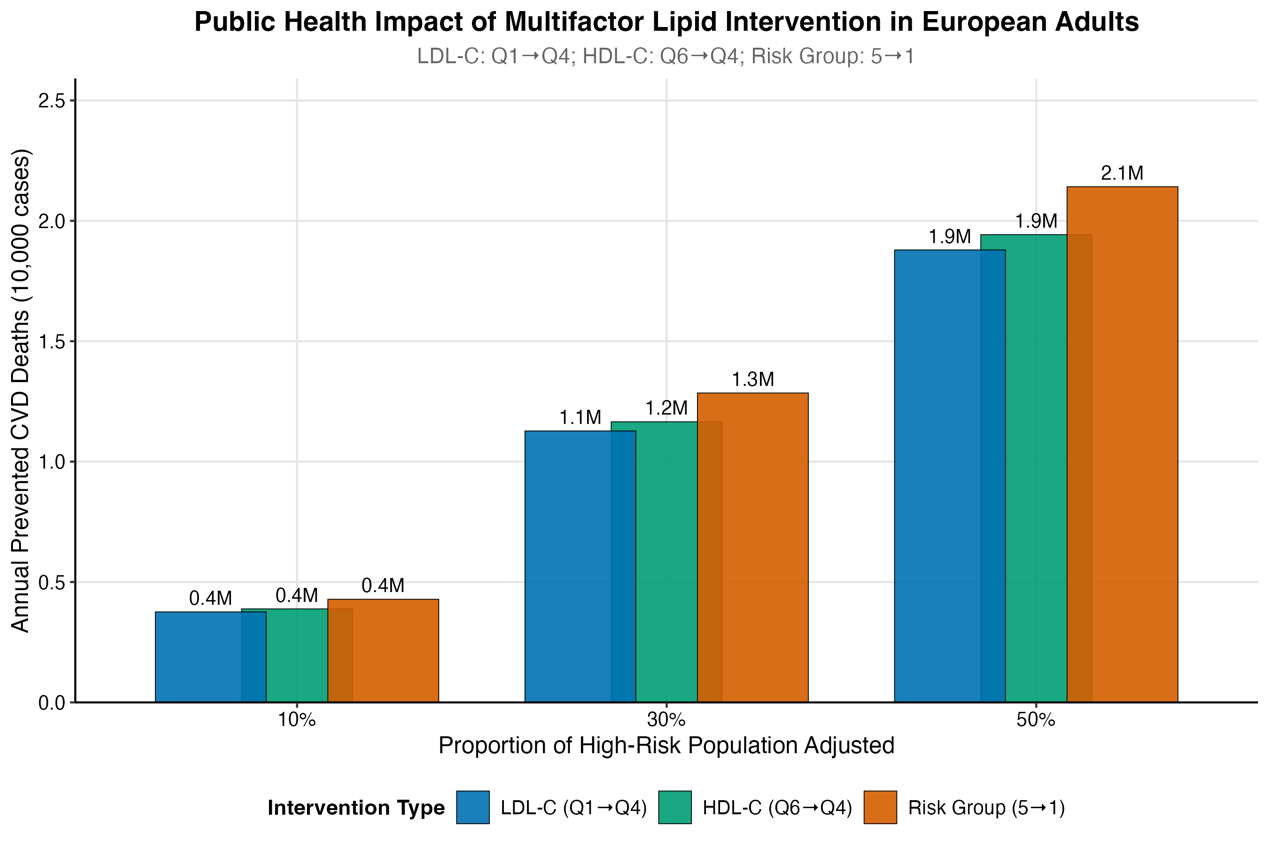


**Figure S4** Public Health Impact of Multifactor Lipid Intervention in European Adults

Based on the WHO (World Health Organisation) European Region's 2024 cvd data report, we estimate that even with a 10% adjustment efficiency, optimising LDL-C, HDL-C, and risk stratification to appropriate thresholds could reduce cvd-related deaths in the European Region by nearly 400,000.

| 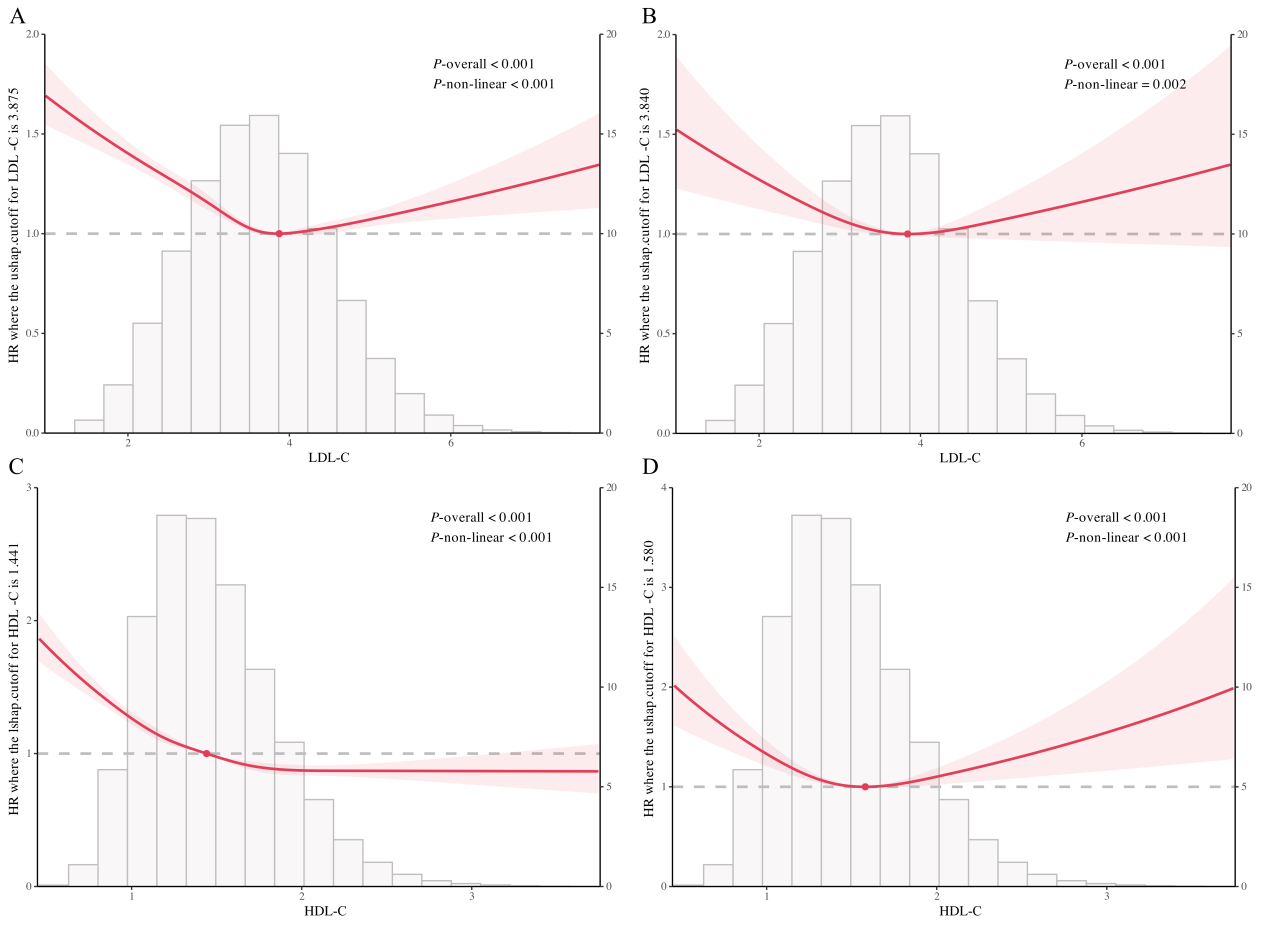 |
| --- |
| **Figure S5 Associations of lipid profile with the risk of CVD incidence and mortality in Cox models with restricted cubic splines after adjustment (excluding participants who died within the first year).**  A: LDL-C with CVD incidence. B: LDL-C with CVD mortality. C: HDL-C with CVD incidence. D: HDL-C with CVD mortality. Model adjusted for sex and age, race, alcohol consumption, smoking status, hypertension, diabetes, and BMI. |

**
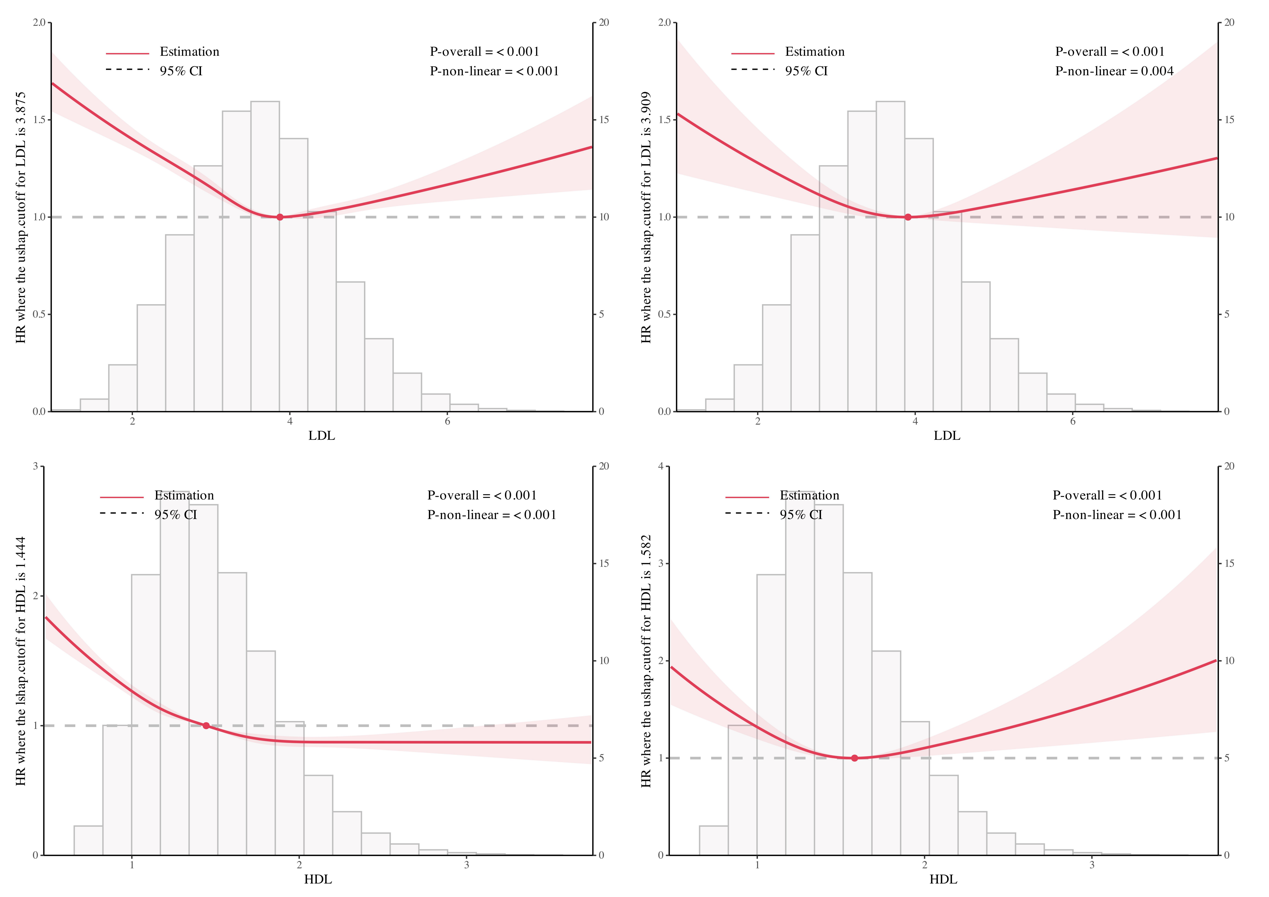
**

**Figure S6Associations of lipid profile with the risk of CVD incidence and mortality in Cox models with restricted cubic splines after adjustment (excluding participants who died within 3 year).**

A: LDL-C with CVD incidence. B: LDL-C with CVD mortality. C: HDL-C with CVD incidence. D: HDL-C with CVD mortality. Model adjusted for sex and age, race, alcohol consumption, smoking status, hypertension, diabetes, and BMI.

**
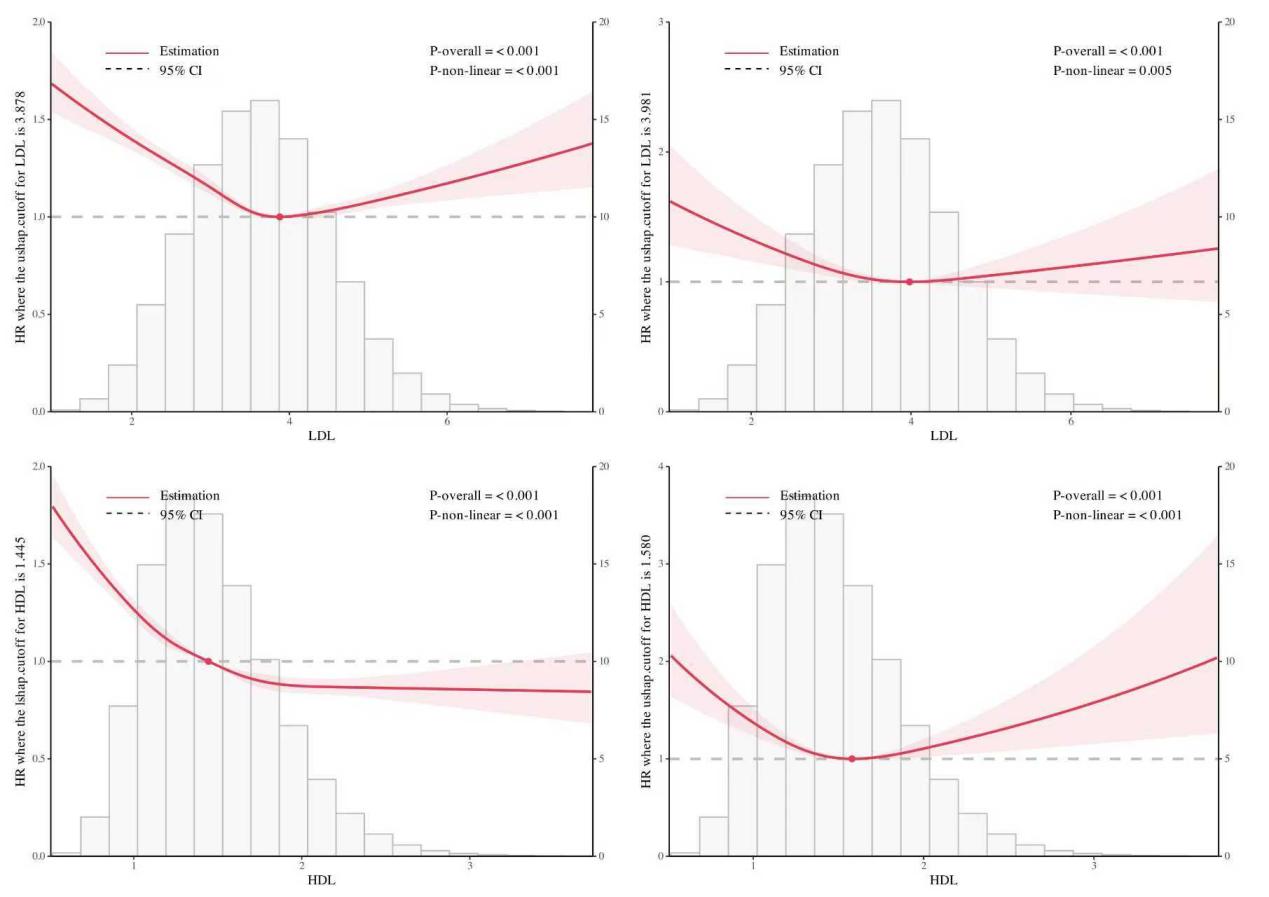
**

**Figure S7 Associations of lipid profile with the risk of CVD incidence and mortality in Cox models with restricted cubic splines after adjustment (excluding participants who died within 5 year).**

A: LDL-C with CVD incidence. B: LDL-C with CVD mortality. C: HDL-C with CVD incidence. D: HDL-C with CVD mortality. Model adjusted for sex and age, race, alcohol consumption, smoking status, hypertension, diabetes, and BMI.

**
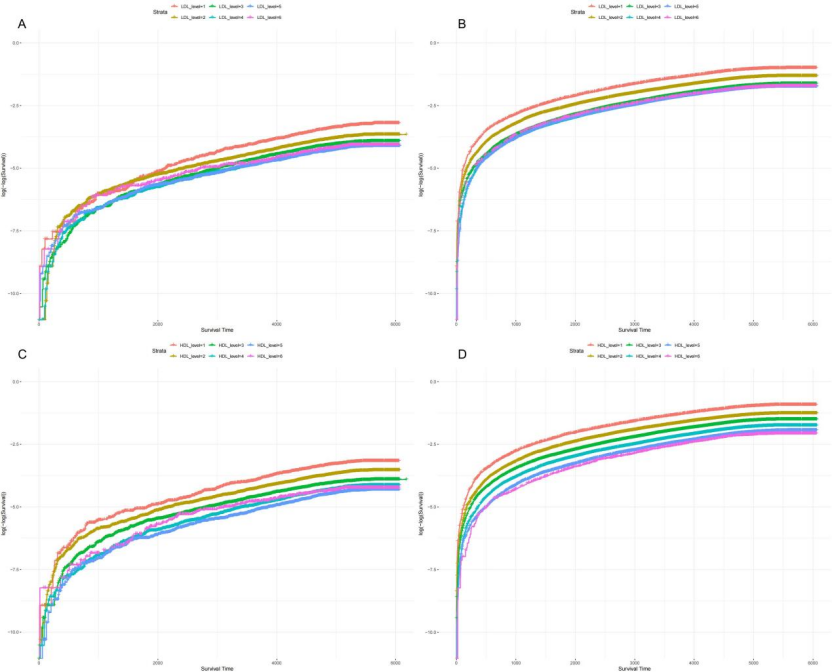
**

**Figure S8** Log-Log Survival Curves (LLS): Proportional Hazards Assumption for LDL-C, HDL-C and CVD Outcomes.

A: LDL-C with CVD mortality. B: LDL-C with CVD incidence. C: HDL-C with CVD mortality. D: HDL-C with CVD incidence.
